# Complete chloroplast genomes of the hemiparasitic genus *Cymbaria*: Insights into comparative analysis, development of molecular markers, and phylogenetic relationships

**DOI:** 10.1002/ece3.11677

**Published:** 2024-07-03

**Authors:** Yang Ma, Jordi López‐Pujol, Dongqing Yan, Zhen Zhou, Zekun Deng, Jianming Niu

**Affiliations:** ^1^ School of Ecology and Environment Inner Mongolia University Hohhot China; ^2^ Botanic Institute of Barcelona (IBB) CSIC‐CMCNB Barcelona Spain; ^3^ Escuela de Ciencias Ambientales Universidad Espíritu Santo (UEES) Samborondón Ecuador; ^4^ Ministry of Education Key Laboratory of Ecology and Resource Use of the Mongolian Plateau Hohhot China; ^5^ Inner Mongolia Key Laboratory of Grassland Ecology and the Candidate State Key Laboratory of Ministry of Science and Technology Hohhot China

**Keywords:** DNA barcoding, hemiparasite, Mongolian medicinal herb, Orobanchaceae, plastome, reductive evolution

## Abstract

The hemiparasitic tribe Cymbarieae (Orobanchaceae) plays a crucial role in elucidating the initial stage of the transition from autotrophism to heterotrophism. However, the complete chloroplast genome of the type genus *Cymbaria* has yet to be reported. In addition, the traditional Mongolian medicine *Cymbaria daurica* is frequently subjected to adulteration or substitution because of the minor morphological differences with *Cymbaria mongolica*. In this study, the complete chloroplast genomes of the two *Cymbaria* species were assembled and annotated, and those of other published 52 Orobanchaceae species were retrieved for comparative analyses. We found that the *Cymbaria* chloroplast genomes are characterized by pseudogenization or loss of stress‐relevant genes (*ndh*) and a unique *rbc*L–*mat*K inversion. Unlike the high variability observed in holoparasites, *Cymbaria* and other hemiparasites exhibit high similarity to autotrophs in genome size, guanine–cytosine (GC) content, and intact genes. Notably, four pairs of specific DNA barcodes were developed and validated to distinguish the medicinal herb from its adulterants. Phylogenetic analyses revealed that the genus *Cymbaria* and the *Schwalbea*–*Siphonostegia* clade are grouped into the tribe Cymbarieae, which forms a sister clade to the remaining Orobanchaceae parasitic lineages. Moreover, the diversification of monophyletic *Cymbaria* occurred during the late Miocene (6.72 Mya) in the Mongol–Chinese steppe region. Our findings provide valuable genetic resources for studying the phylogeny of Orobanchaceae and plant parasitism, and genetic tools to validate the authenticity of the traditional Mongolian medicine “*Xinba.*”

## Graphical abstract

Specific DNA barcodes were developed and validated from the *Cymbaria* chloroplast genomes to distinguish the traditional Mongolian medicine *Cymbaria daurica* from its adulterant *Cymbaria mongolica*.

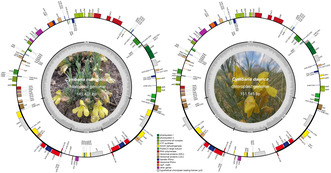

## 1 INTRODUCTION

Chloroplasts are semiautonomous organelles that carry out photosynthesis in green plants. Chloroplast genome typically exhibits maternal inheritance, which differentiates it from the biparentally inherited nuclear genome (Birky, 1995). Generally, autotrophic plants are highly conserved in the chloroplast genomes (Wicke et al., 2011). By contrast, heterotrophic plants including mycoheterotrophs and parasites exhibit varying degrees of degeneration in their chloroplast genomes (Graham et al., 2017). The family Orobanchaceae is recognized as the largest single family of parasites, consisting of 101 genera and over 2100 species with autotrophic, hemiparasitic, and holoparasitic lifestyles (Byng et al., 2016; Nickrent, 2020). Orobanchaceae is thus considered an excellent model to study the evolution of parasitic plants (Westwood et al., 2012). Most previous studies have focused on autotrophs and holoparasites (Xiaoqing Liu et al., 2019; Wicke & Naumann, 2018; Zeng et al., 2017). However, limited investigations have been done on hemiparasites (Li et al., 2021; Zhang, Xu, et al., 2020). The hemiparasitic tribe Cymbarieae comprises approximately 20 species in five genera, and their study plays a crucial role in elucidating the initial stage of the transition from autotrophism to heterotrophism (Schneeweiss, 2013). Cymbarieae was originally thought to be sister to various parasitic lineages in the family Orobanchaceae (Bennett & Mathews, 2006; McNeal et al., 2013), while analysis of the tribe Orobancheae suggests that this original classification merits reconsideration (Li et al., 2019). Furthermore, no systematic comparative analysis of chloroplast genomes of various groups within the tribe Cymbarieae has been conducted to date, although chloroplast genomes of *Schwalbea americana* (Wicke et al., 2013) and *Siphonostegia chinensis* (Gao et al., 2019; Jiang et al., 2022) have been previously published.

*Cymbaria* L. sensu stricto (i.e., excluding *Cymbochasma* Endl.) is the type genus of the tribe Cymbarieae and has only two facultative hemiparasites, *C. mongolica* Maxim. and *C. daurica* L. (Zhao, 1999). *Cymbaria mongolica* is endemic to the Loess Plateau of China, being distributed in Northwest China. *C. daurica* L. is a characteristic species of Mongolian Plateau steppe, being distributed in North China, Northeast China, Mongolia, and Eastern Siberia of Russia. Both species are yellow‐flowered short perennial herbs and show an overlapping distribution across southern Inner Mongolia Autonomous Region, northern Ningxia, Shaanxi, Shanxi, and Hebei Provinces in China (Hong et al., 1998). *Cymbaria daurica* has a chromosome count of 2*n* = 32 (Marhold et al., 2023). According to the *Chinese Pharmacopoeia* (Commission of Chinese Pharmacopoeia, 2015) and the *Mongolian Medicinal Materials Standard* (Inner Mongolia Ministry of Health, 1987), *C. daurica* is the only original plant species for elaborating the traditional Mongolian medicine, whereas *C. mongolica* has no medicinal value. The traditional Mongolian medicine *C. daurica* is referred to as “*Kanba‐Arong*” in Mongolian and “*Xinba*” in Chinese. It is widely used for treating several diseases, including pruritus, psoriasis, fetotoxicity, impetigo, and diabetes (Zhang et al., 2013). Its historical application dates back to the middle of the eighteenth century, as it was already included in the Mongolian pharmaceutical classic known as “*RenYaoBaiJingJian*.” Subsequently, it has been extensively documented across the folk medicinal literature (as illustrated in Figure S1). The dried whole plant is also included in several classical herbal formulations, such as “*Siweixinbasan*” and “*Baweixinbasan*.” Previous studies have shown that *C. daurica* contains 177 chemical components (Wu et al., 2020), being the most common flavonoids (Li et al., 2014) and iridoid glycosides (Wang et al., 2018). In modern pharmacological research, the herb has received much recent attention owing to its antidiabetic (Gong et al., 2020; Shi, Chen, et al., 2023; Shi, Li, et al., 2023) and anti‐inflammatory (Guo et al., 2017; Huang et al., 2023) properties. However, either accidental or intentional, the adulteration and substitution of *C. daurica* frequently occurs in China and Mongolia. This growing problem has been attributed to the profit‐driven individuals seeking improper financial gains from the cheaper substitute, morphologically similar *C. mongolica*, resulting in confusion either during the initial identification process or in the course of manufacture (Hu, 2018; Liang et al., 2016; Zhang et al., 2013). This issue has an unforeseeable subsequent effect on the herb “*Xinba*,” jeopardizing its clinical use, potency, and safety.

Currently, morphological microscopic characteristics are used to identify these two *Cymbaria* species (Wang et al., 2012). However, such method relies on the anther morphology and should be carried out by specialists, who are required to obtain accurate identifications. In recent years, researchers have shifted their focus from the traditional morphological and chemical identification to using DNA‐based molecular markers as a precise method to assess the authenticity of medicinal herbs. DNA barcodes have become particularly popular for identifying species of Chinese medicinal herbs owing to their accuracy and speed (Zhu et al., 2022). The universal barcodes include ITS, *mat*K, *rbc*L, and *trn*H–*psb*A, either individually or in combination (CBOL Plant Working Group, 2009; China Plant BOL Group et al., 2011). However, these barcodes might be ineffective for complex taxonomic groups, especially for recently evolved and closely related taxa, because sufficient genetic variation is lacking (Xu, Zhao, et al., 2022). Therefore, it is really necessary to develop specific DNA barcodes to distinguish the herb *C. daurica* from its adulterant *C. mongolica*, which will overcome the need of doing the (morphological) identification during the species short flowering period and by specialized personnel.

Here, we provide the newly assembled and annotated chloroplast genomes of both *Cymbaria* species and conduct a comparative analysis including the chloroplast genomes of other 52 Orobanchaceae species. Our specific objectives were to (1) characterize *Cymbaria* chloroplast genomes, (2) develop the specific molecular markers as DNA barcodes, and (3) infer the phylogeny and divergence time within the *Cymbaria* genus and regarding the family Orobanchaceae. These findings will provide key insights into the taxonomic identification, phylogenetic placement, and reductive evolution of hemiparasitic genus *Cymbaria*, and valuable genetic tools to validate the authenticity of the traditional Mongolian medicine “*Xinba*.”

## 2 MATERIALS AND METHODS

### 2.1 Sequencing, assembly, and annotation

Wild *Cymbaria mongolica* and *C. daurica* plants were collected from Binzhou, Shaanxi Province (35°14′38.63″ N, 108°13′06.11″ E) and Xilingol, Inner Mongolia Autonomous Region (43°28′13.92″ N, 116°47′07.76″ E) in China. One sample of young fresh leaves per species was collected in a liquid nitrogen tank. The habitat and altitude were also recorded. Voucher specimens were stored in the Herbarium of Inner Mongolia University, with accession numbers MYCM22052703 and MYCD22052903 for *C. mongolica* and *C. daurica*, respectively. Total genomic DNA was extracted using the modified cetyltrimethylammonium bromide (CTAB) method (Allen et al., 2006). The DNA Library with average insert sizes of 350 bp (base pairs) was prepared by a NEBNext® Ultra II™DNA Library Prep Kit following the manufacturer's instructions (Illumina Inc., San Diego, CA, USA), and paired‐end libraries (150 bp) were sequenced using an Illumina HiSeq 2500 System. The raw reads were filtered by NGS QC Toolkit v2.3.3 (Patel & Jain, 2012) to obtain high‐quality reads. Contigs were de novo assembled through SPAdes v3.14.0 (Bankevich et al., 2012) using the parameters of “‐k 21,33,55,77,99,127 ‐careful.” Final assembly maps were further visualized using Bandage (Wick et al., 2015). Annotations were performed using the GeSeq online tool (Tillich et al., 2017) with default settings and manually adjusted in Geneious v9 (Kearse et al., 2012). After identifying the boundaries by BLAST (Basic Alignment Local Search Tool), the sequences were submitted to GenBank and visualized as a circular map using the OrganellarGenomeDRAW (OGDRAW) tool v1.3.1 (Greiner et al., 2019).

### 2.2 Comparative analysis of chloroplast genomes

A comparative analysis of Orobanchaceae chloroplast genomes was conducted including eight autotrophs, 21 hemiparasites, and 25 holoparasites. The genome sizes, guanine–cytosine (GC) contents, and intact genes were investigated using PhyloSuite v1.2.1 (Zhang, Gao, et al., 2020) and drawn with RadarMap package in R software (RStudio Team, 2015). A heatmap of genes in the chloroplast genome was generated using TBtools‐II v1.120 (Chen et al., 2023). The PAML (Phylogenetic Analysis by Maximum Likelihood) package v4.0 was used to calculate the nonsynonymous (*Ka*) and synonymous (*Ks*) substitution rates and their ratio (ω = *Ka*/*Ks*) (Yang, 2007). Contractions and expansions of the boundaries were detected using the online IRscope software (Amiryousefi et al., 2018). The Mauve program v2.3.1 was used to align sequences against the *Schwalbea americana* L. reference sequence (Darling et al., 2010). The relative synonymous codon usage (RSCU) and the effective number of codons (ENC) were calculated by CodonW v1.4.2 (http://codonw.sourceforge.net/) and the Emboss online website CUSP module (http://imed.imed.ucm.es/EMBOSS/), respectively. The online REPuter software tool (Kurtz et al., 2001) was employed to detect repeat sequences. Simple sequence repeats (SSRs) were identified by MISA‐web (Beier et al., 2017).

### 2.3 Development and validation of DNA barcodes

Structural comparisons were conducted using mVISTA (Frazer et al., 2004) with *C. mongolica* as a reference. Sliding window analysis was conducted to identify hypervariable regions using DnaSP (DNA Sequence Polymorphism) v5.10 (Librado & Rozas, 2009). Primer3web v4.1.0 (Kõressaar et al., 2018) was used to design DNA barcoding primers based on the hypervariable regions, and these were verified using seven individuals from different regions of each species. Polymerase chain reaction (PCR) was conducted in 25 μL reaction mixtures with 12.5 μL of 2 × EasyTaq PCR SuperMix, 1.0 μL of each primer (0.4 μM), 1 μL of template DNA, and 9.5 μL of double‐distilled water (ddH_2_O). A SimpliAmp™ Thermal Cycler (Applied Biosystems, Carlsbad, CA, USA) was used to conduct all PCRs with the following thermal cycling conditions: 94°C for 5 min; 30 cycles of 94°C for 30 s, a specific annealing temperature (Tm) for 30 s, and 72°C for 30 s; and 72°C for 10 min. Agarose gel electrophoresis (1.5%) was used to visualize PCR products. The DNA fragments were purified and sequenced by Biomarker Technologies Co., Ltd. (Beijing, China).

### 2.4 Phylogenetic analyses and divergence time estimation

To identify early divergence events within Orobanchaceae, phylogeny was inferred from 54 Orobanchaceae species with *Tectona grandis* L. f. (GenBank accession number: NC_020098) and *Paulownia tomentosa* (Thunb.) Steud. (GenBank accession number: MK875778) as outgroups. Phylogenetic trees were generated using three datasets: (1) complete chloroplast genome sequences; (2) coding DNA sequences (CDSs); and (3) shared CDSs (*rps*2, *rps*7, *rps*8, *rps*14, *rpl*2, *rpl*16, *rpl*36, and *mat*K). The standard phylogenetic workflow was conducted in PhyloSuite v1.2.2 (Zhang, Gao, et al., 2020) as follows: (1) to align sequences with MAFFT (multiple sequence alignment using Fast Fourier Transform) v7.313 (Katoh & Standley, 2013), (2) to achieve optimization regions with Gblocks v0.91b (Castresana, 2000), (3) to achieve concatenation datasets with the Concatenate Sequence function integrated in PhyloSuite v1.2.2 (Zhang, Gao, et al., 2020), (4) to select optimal partitioning scheme with PartitionFinder2 v2.1.1 (Lanfear et al., 2017), and (5) to infer phylogeny using Maximum likelihood (ML) in IQ‐TREE v1.6.8 (Nguyen et al., 2015) and Bayesian inference (BI) in MrBayes v3.2.6 (Drummond et al., 2012). Phylogenetic trees were visualized using iTOL (Interactive Tree of Life) v6 (Letunic & Bork, 2021).

BEAST (Bayesian Evolutionary Analysis Sampling Trees) v1.7 (Drummond et al., 2012) was used to estimate divergence times based on the shared concatenated CDSs. As no reliable fossil record is available, the crown age for Orobanchaceae and Pedicularideae was constrained to 56 ± 10 Mya (million years ago) (obtained from TimeTree 5) (Kumar et al., 2022) and to 35 ± 10 Mya (Yu et al., 2018), respectively. The results of PartitionFinder were used to determine the nucleotide substitution model of the unlinked subsets. The following parameters were used in BEAUti (Bayesian Evolutionary Analysis Utility) software: “Lognormal relaxed clock (Uncorrelated)” for “Clock model” and “Speciation: Yule Process” for “Tree model.” Three independent Markov chain Monte Carlo (MCMC) chains were carried out under the same parameters. Each MCMC was run for 50,000,000 generations, and sampling was conducted every 5000 generations. Convergence was evaluated using Tracer v1.6 (http://beast.bio.ed.ac.uk/Tracer). The LogCombiner program was used to combine the three log files. The TreeAnnotator program was used to generate the maximum clade credibility (MCC) tree. The effective sample sizes in the combined three log outputs were greater than 200, which indicated that the MCMC sampling was adequate. FigTree v1.4.3 (Rambaut, 2017) was used to visualize the maximum clade credibility (MCC) tree with 95% highest posterior density (HPD) intervals.

## 3 RESULTS

### 3.1 Characteristics of *Cymbaria* chloroplast genomes

**FIGURE 1:**
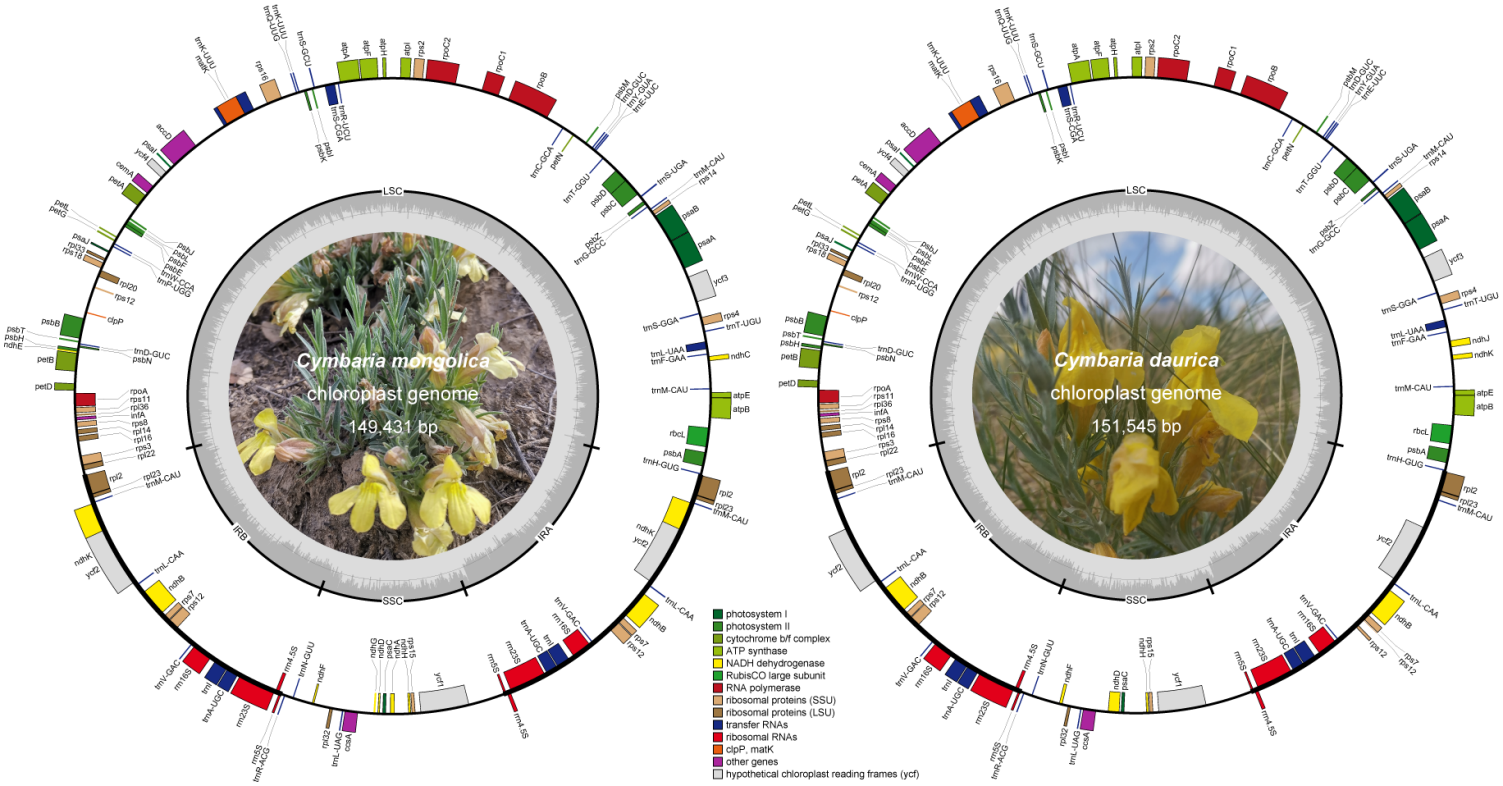
Circular maps of *Cymbaria* chloroplast genomes. Left, *C. mongolica*; right, *C. daurica*.

Illumina sequencing yielded more than 20 million bp clean reads. The chloroplast genomes of *Cymbaria mongolica* and *C. daurica* displayed a typical quadripartite structure with sizes of 149,431 bp (38.0% GC content) and 151,545 bp (38.2% GC content), respectively (Figure 1). Each chloroplast genome comprised a large single‐copy region (LSC, 86,595 bp and 87,376 bp), a small single‐copy region (SSC, 16,962 bp and 17,825 bp), and two inverted repeat regions (IR, 22,937 bp and 23,172 bp). The GC content in the IR region (44.1% in both species) was higher than those in the LSC (35.9%–36.2%) and SSC (32.2%–32.7%) regions.

The chloroplast genome sequences of *C. mongolica* and *C. daurica* contained 105 and 100 intact genes, respectively (Figure 1). *Cymbaria* contained 26 transfer RNA (tRNA) and four ribosomal RNA (rRNA) genes, while the number of protein‐coding genes (PCGs) and pseudogenes varied between the two species (Table S1). In contrast to the autotrophic *Lindenbergia philippensis*, *C. mongolica* encoded 75 PCGs, and one pseudogene and lacked seven genes, while *C. daurica* encoded 70 PCGs, and three pseudogenes and lacked 10 genes. Among the 15 intron‐containing genes, 12 genes contained one intron, and the remaining three genes harbored two introns (Table S1). In addition, purifying or neutral selection was detected on all PCGs, with the exception of *ycf*2, which was under positive selection. No pronounced differences were detected in the boundary regions. An inversion of large gene blocks (*rbc*L–*mat*K) was identified in the LSC region according to the Mauve alignment (Figure S2). Four pairs of palindromic repeats (123, 67, 48, and 37 bp) and three pairs of palindromic repeats (190, 152, and 121 bp) were detected at both ends of the inverted region of *C. mongolica* (54,654 bp size) and *C. daurica* (55,904 bp size), respectively.

**FIGURE 2:**
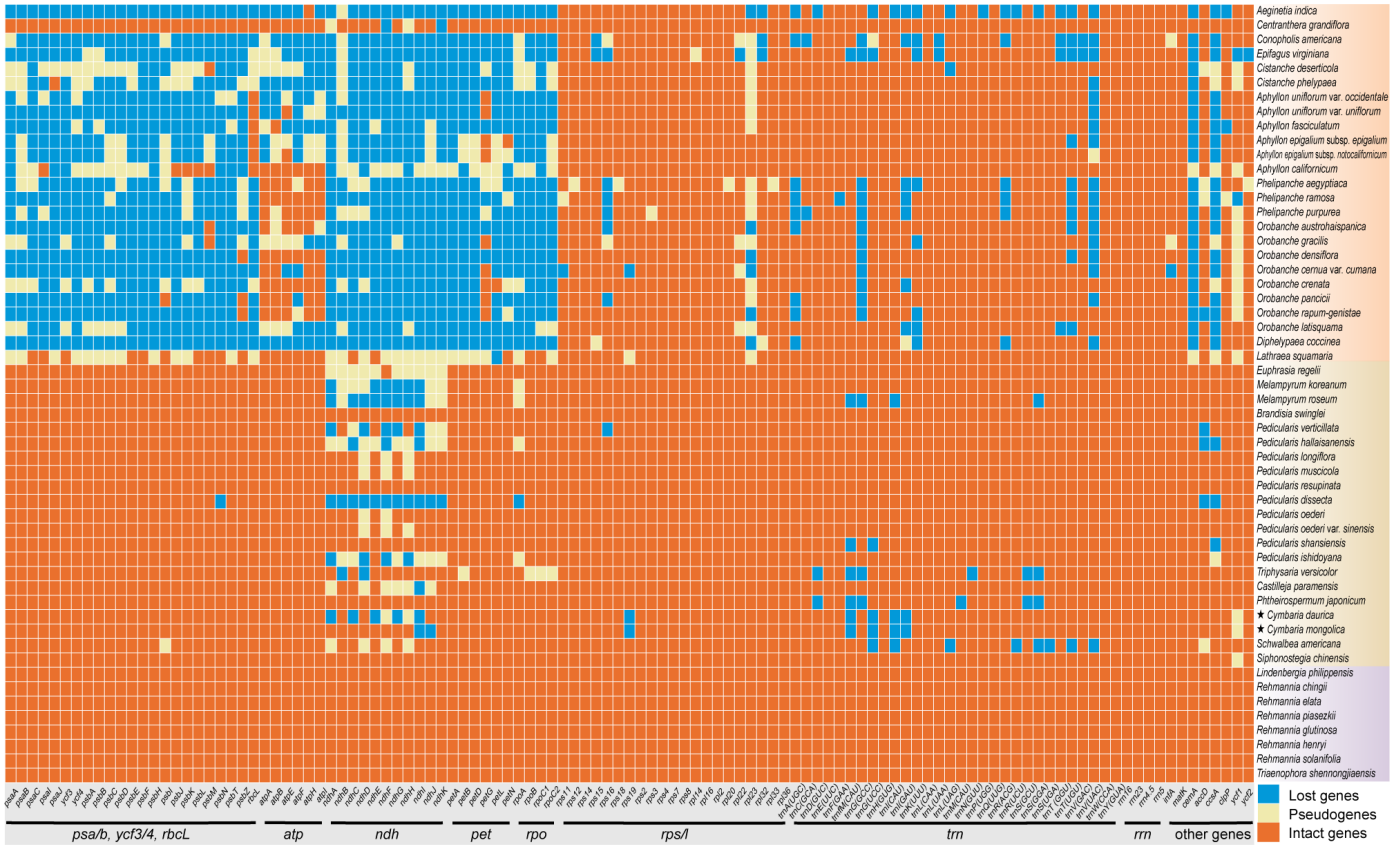
Heatmap depicting the chloroplast gene content across 54 Orobanchaceae species. Blocks in orange, linen, and blue indicate intact genes, pseudogenes, and lost genes, respectively. Background colors in purple, brown, and orange indicate autotrophs, hemiparasites, and holoparasites, respectively.

We estimated the sequence divergence and gene content for 54 chloroplast genomes within the family Orobanchaceae (Table S2). The genome sizes of the autotrophs (153,622–155,319 kb, mean: 154,213 kb) and hemiparasites (142,733–160,910 kb, mean: 151,152 kb) were similar; however, the genome sizes of the autotrophs were higher than those of the holoparasites (45,673–150,504 kb, mean: 87,505 kb) (Figure S3). The GC content was lower in holoparasites (mean: 34.93%) than in hemiparasites (38.25% on average) and autotrophs (mean: 37.90%). Autotrophs contained all intact genes; the number of intact genes was lower in hemiparasites than in autotrophs, and this decrease primarily stemmed from the pseudogenization/loss of *ndh* genes (Figure 2). Moreover, non‐functionalization and gene loss were observed in most photosynthetic genes (e.g., *psa*/*psb*, *ycf*3/4, *ndh*, and *cem*A) in holoparasites.

### 3.2 Codon usage bias

**FIGURE 3:**
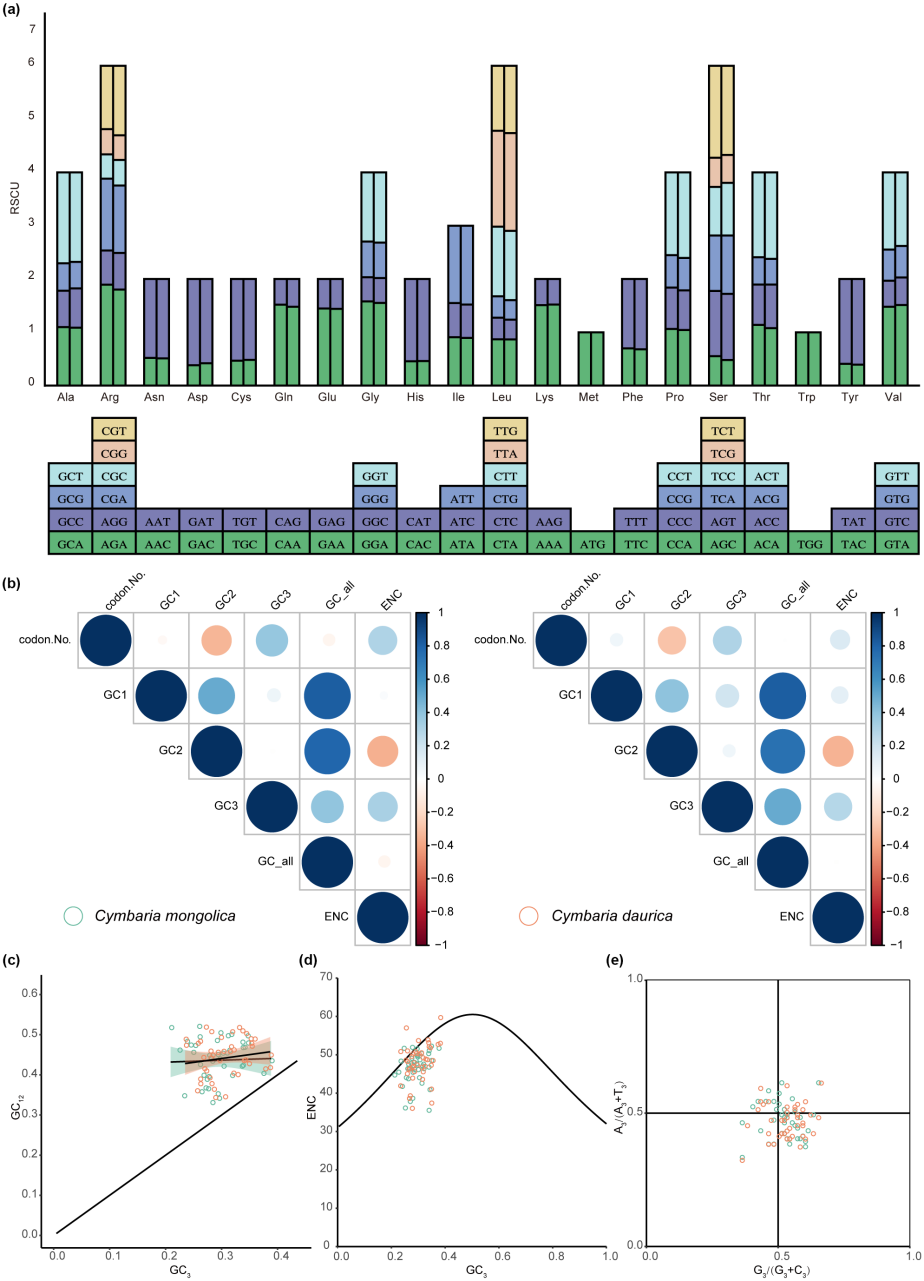
Codon usage bias of *Cymbaria* chloroplast genomes. (a) RSCU. (b) Correlation heatmap. (c) Neutral plot analysis. (d) PR2‐plot analysis. (e) ENC plot analysis. Circle colors of green and orange represent *C. mongolica* (left) and *C. daurica* (right), respectively.

The PCGs in *Cymbaria mongolica* and *C. daurica* comprised 18,482 and 15,896 codons, respectively. Leucine (Leu, 10.50% and 10.62%) was the most common amino acid, and cysteine (Cys, 1.09% and 1.07%) was the least common amino acid. A total of 30 codons (RSCU > 1) were A/T‐ending codons, with the exception of UUG (Figure 3a). The range of the ENC in *C. mongolica* and *C. daurica* was 35.24–56.04 and 35.75–59.41, respectively, which suggested weak codon usage bias. ENC was significantly negatively correlated with GC content at the second position of codons (GC2) in both species; a significant positive correlation between ENC and GC content at the third position of codons (GC3) was only detected in *C. mongolica* (Figure 3b). There was no significant correlation between GC3 and GC content at the first two positions of codons (GC12) according to neutral plot analysis (Figure 3c); the regression coefficient was 0.049 and 0.193 in *C. mongolica* and *C. daurica*, respectively. Most genes were below and around the standard curve according to ENC plot analysis (Figure 3d), and the ENC ratio was from 0.05 to 0.15. Parity rule 2 (PR2)‐plot analysis (Figure 3e) showed that T > A and G > C in the base usage frequency. A total of 16 codons were identified as preferred codons, of which 10 were shared by the two species.

### 3.3 Repetitive sequence variation

**FIGURE 4:**
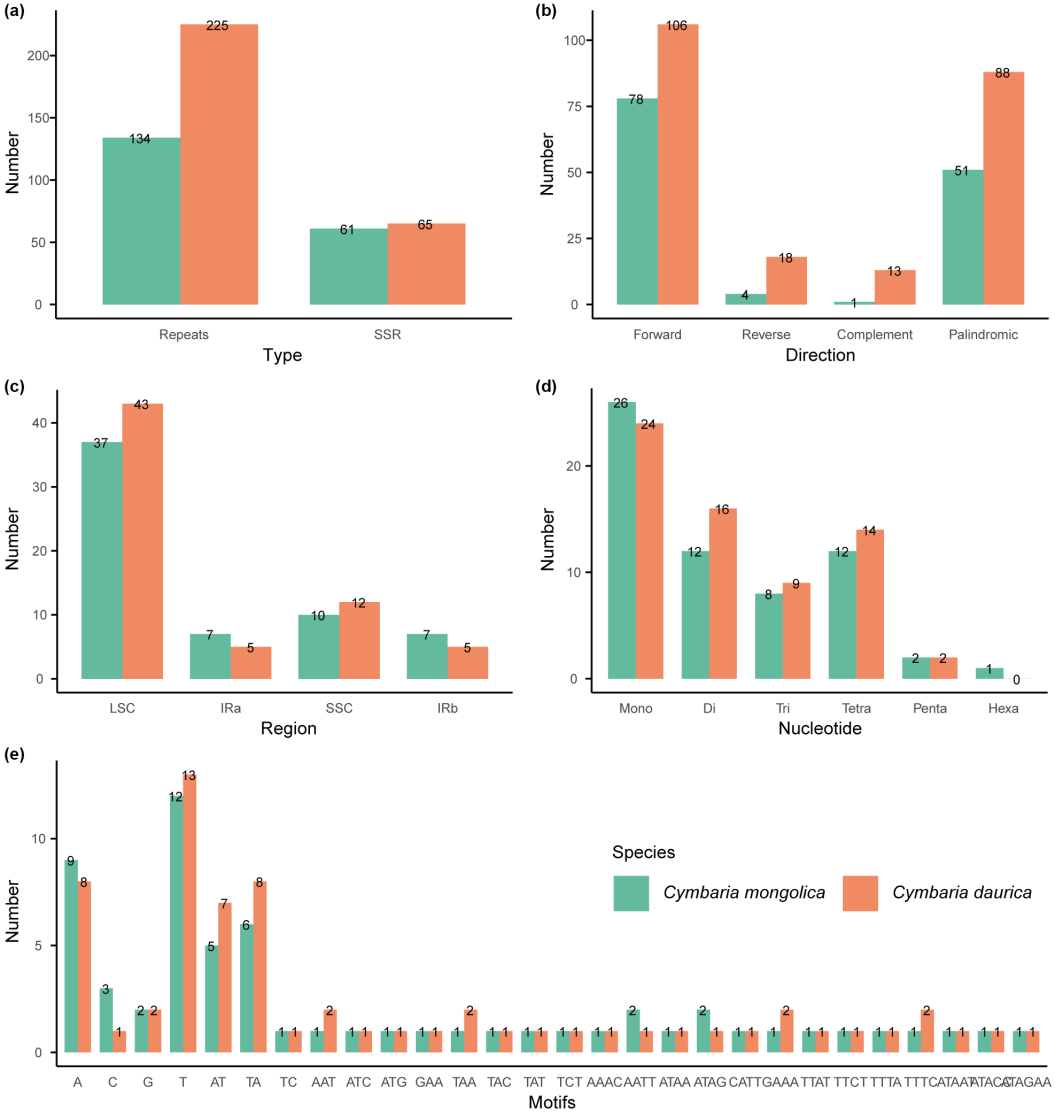
Comparison of the repeats and SSRs in *Cymbaria* chloroplast genomes. (a) Repeats and SSRs. (b) Repeat types. (c) Occurrences of SSRs. (d) SSR types. (e) SSR motif types. Bar colors of green and orange correspond to *C. mongolica* (left) and *C. daurica* (right), respectively.

A total of 134 repeats, consisting of 78 forward, four reverse, one complementary, and 51 palindrome repeats, were detected from *Cymbaria mongolica* chloroplast genome. Meanwhile, a total of 225 repeats, including 106 forward, 18 reverse, 13 complementary, and 88 palindrome repeats, were identified from *C. daurica* chloroplast genome (Figure 4a,b). The size of approximately 90% of the repeats ranged from 30 bp to 70 bp. *C. mongolica* and *C. daurica* contained 61 and 65 SSRs, and most of them were present in the LSC region (37 and 43 SSRs), respectively (Figure 4a,c). Hexa‐nucleotide SSRs were only detected in *C. mongolica*; the remaining SSRs were identified in both species (Figure 4d). Mono‐nucleotides were the most plentiful, followed by di‐ and tetra‐nucleotides. The mononucleotide motifs A/T had the highest proportion, accounting for 34.4% in *C. mongolica* and 32.3% in *C. daurica* (Figure 4e).

### 3.4 Development and validation of DNA barcodes

**FIGURE 5:**
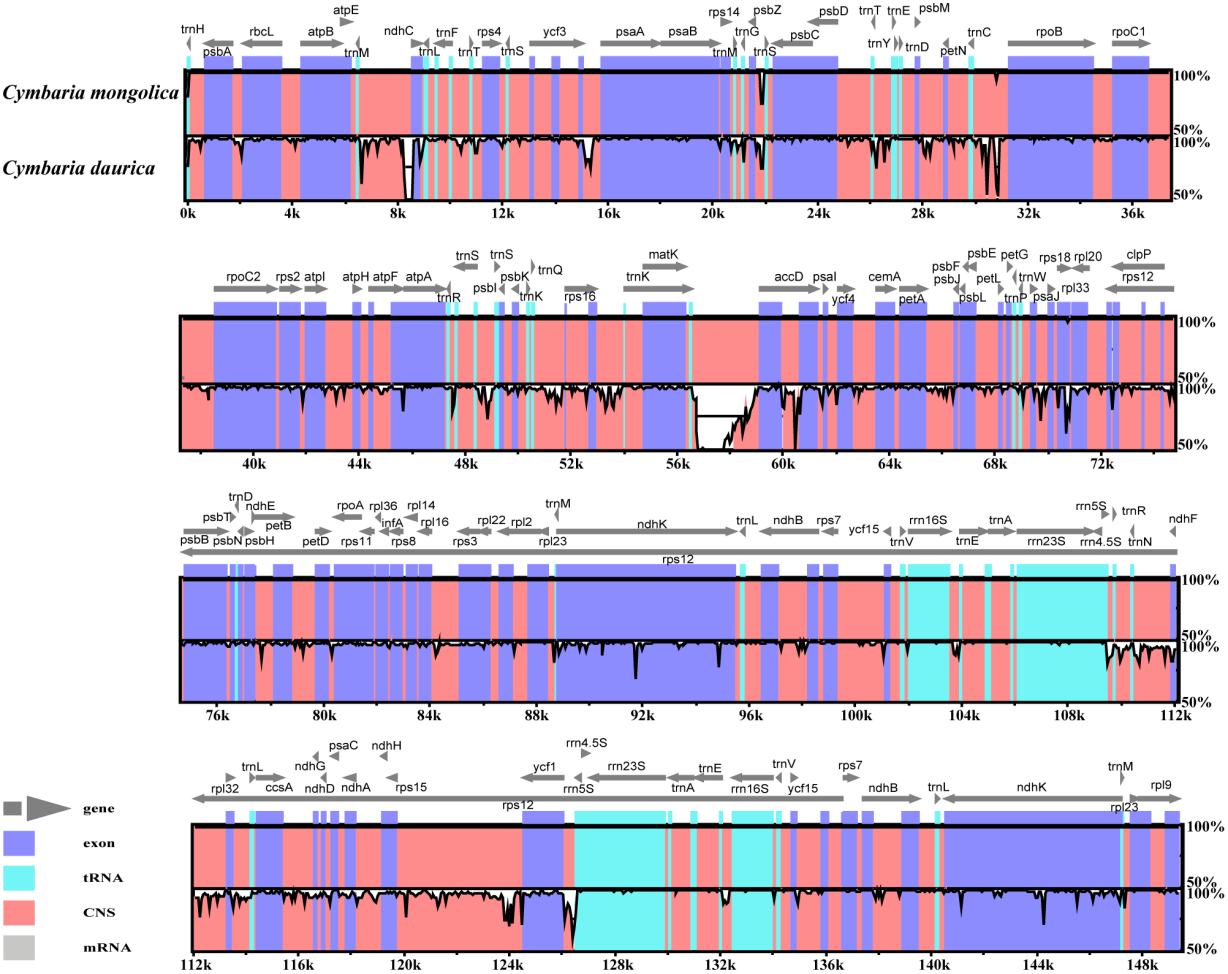
Sequence alignment of *Cymbaria* chloroplast genomes.

**FIGURE 6:**
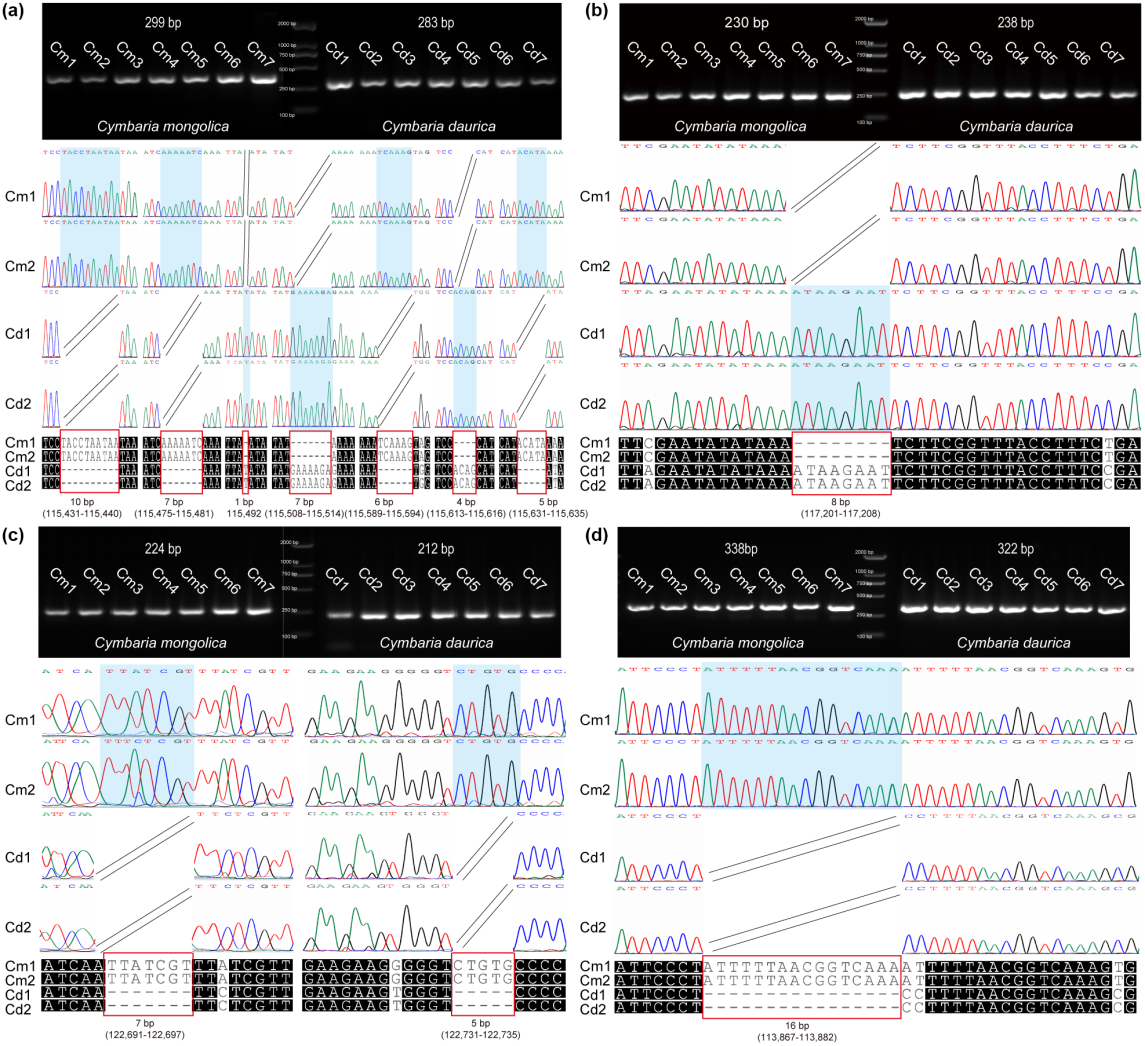
Agarose gel electrophoresis, chromatogram, and alignment of sequences obtained via Sanger sequencing of the amplified DNA barcodes. The indel makers include (a) CymN1, (b) CymN2, (c) CymY, and (d) CymR. The lanes correspond to the PCR products amplified from seven individuals of *C. mongolica* (left) and *C. daurica* (right). The red squares correspond to the indel regions.

High conservation with some degree of divergence was observed in the two chloroplast genomes (Figure 5). Most of the sequence differences were observed in the non‐coding regions. Nucleotide diversity (Pi) was 0.02099, and higher divergence was observed in the LSC and SSC regions (Figure S4). We also detected several divergence hotspot regions (Pi > 0.05), including *trn*M–CAU–*ndh*C, *psa*A, *mat*K, *acc*D–*psa*I, *ycf*4–*cem*A, *rpl*32–*trn*L–UAG, *ndh*D–*ndh*G, *rps*15–*ycf*1, *rrn*23S, and *trn*A–UGC–*trn*E–UUC. We designed four specific DNA markers (*CymN1*, *CymN2*, *CymY*, and *CymR*), and they were validated using sequences from different regions from seven individuals of each species (Tables S3 and S4). DNA fragments varying in length were obtained from *Cymbaria mongolica* and *C. daurica* using each primer, and both species could be distinguished via agarose gel electrophoresis (Figure 6). The original uncropped image is presented in Figure S5. Likewise, in the results of sequencing alignment we observed that each barcode had at least one indel locus and several single nucleotide polymorphism (SNP) loci (Figures S6–S9). Overall, these findings suggest that the four pairs of DNA barcodes could be used to distinguish among *Cymbaria* species.

### 3.5 Phylogenetic relationships and divergence times

**FIGURE 7:**
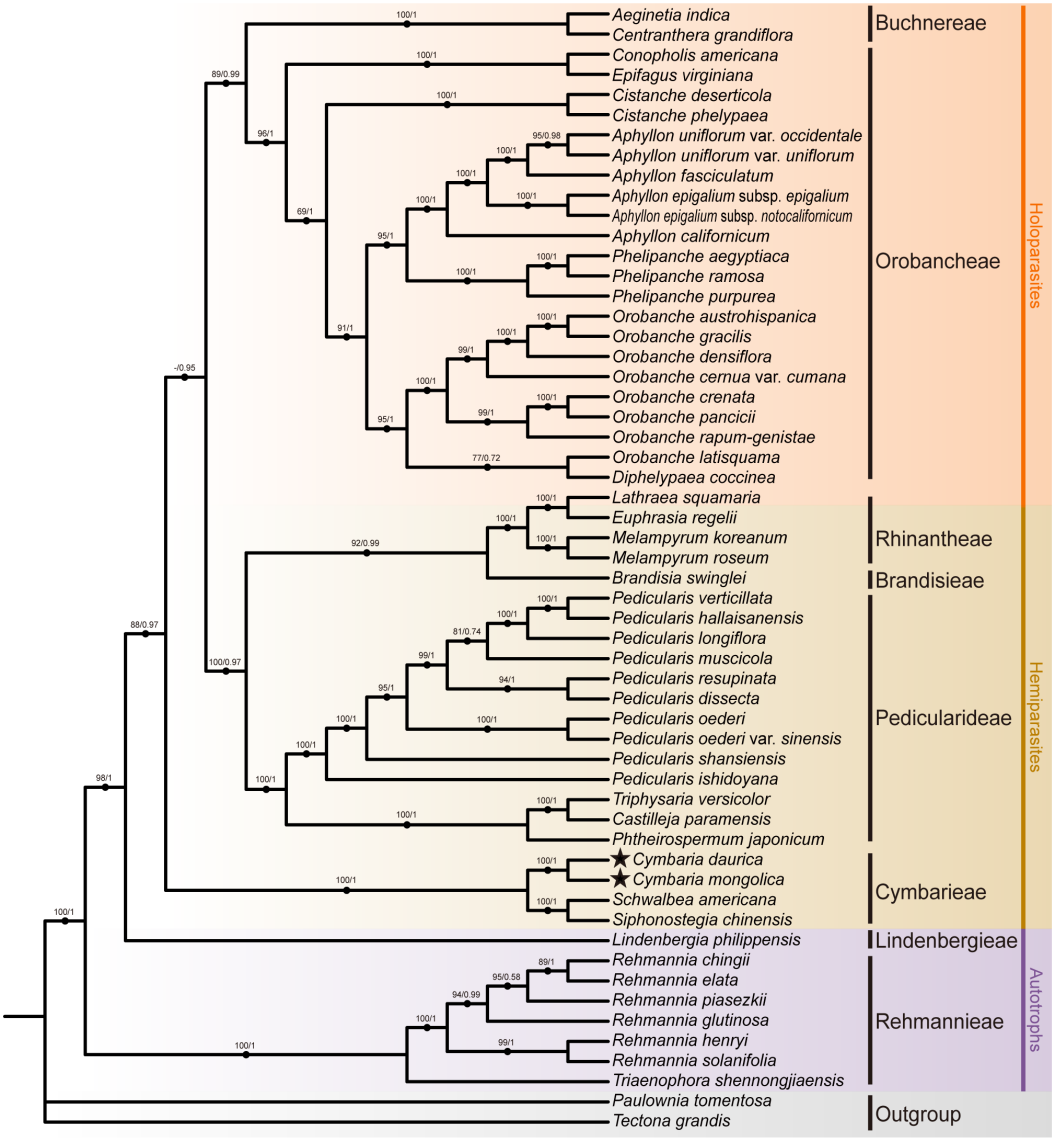
Phylogenetic relationship inferred from ML and BI based on shared protein‐coding genes of 54 Orobanchaceae species. Numbers above the branches indicate bootstrap values (left) and posterior probabilities (right). Background colors of gray, purple, brown, and orange indicate outgroups, autotrophs, hemiparasites, and holoparasites, respectively. The stars (★) indicate the two newly sequenced *Cymbaria* species.

**FIGURE 8:**
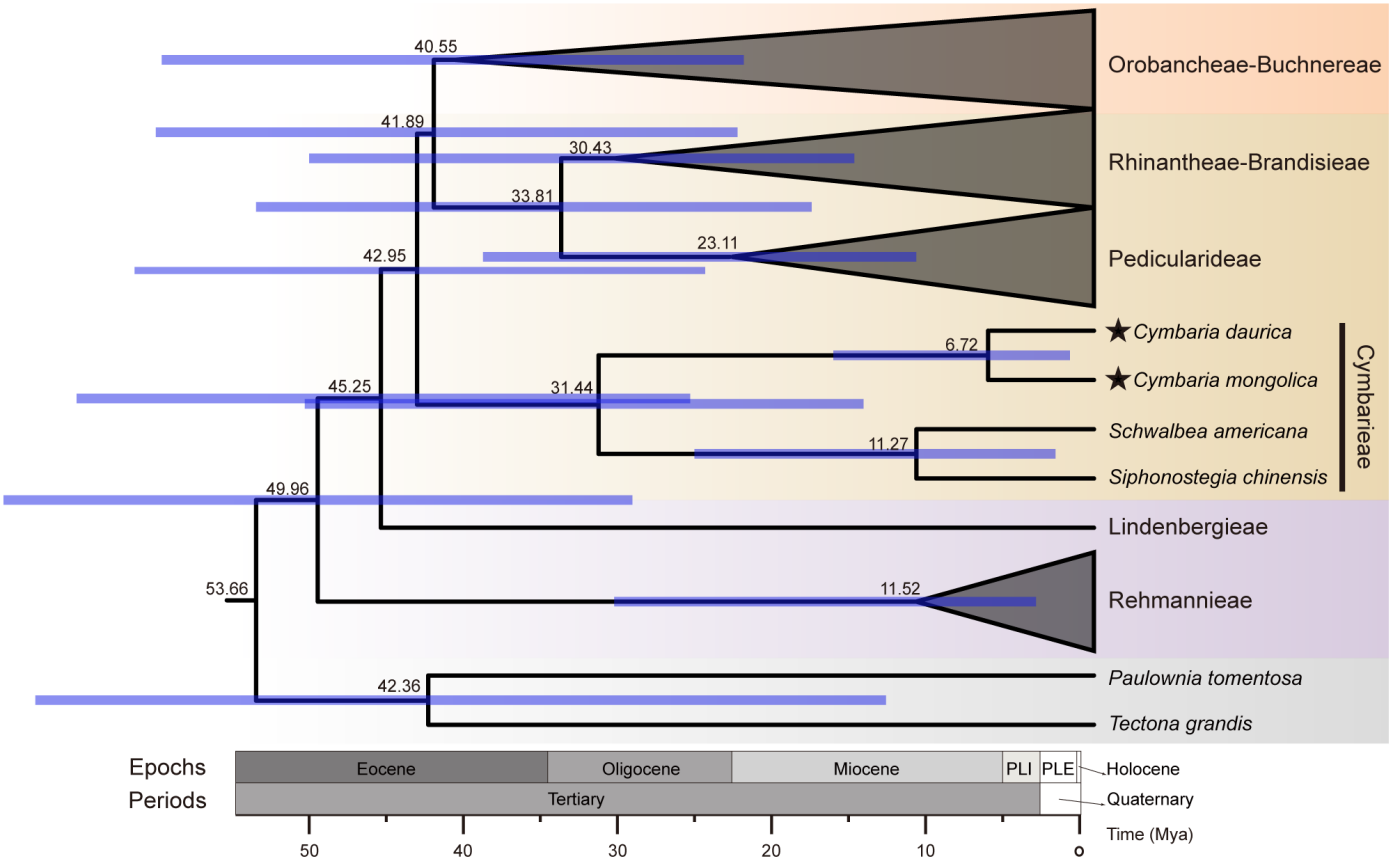
Divergence time estimated from the MCC tree in BEAST. Node numbers indicate mean divergence times (above). Node bars represent the 95% HPD interval (blue bar). Background colors of gray, purple, brown, and orange indicate outgroups, autotrophs, hemiparasites, and holoparasites, respectively. The stars (★) indicate the two newly sequenced *Cymbaria* species.

Maximum likelihood (ML) and Bayesian inference (BI) phylogenetic analyses yielded highly consistent topologies for the three datasets (Figure 7; Figures S10–S13). The monophyly of Orobanchaceae was strongly supported. The tribes Rehmannieae, Lindenbergieae, Cymbarieae, Pedicularideae, Brandisieae, Rhinantheae, Orobancheae, and Buchnereae corresponded to well‐supported clades. The hemiparasitic tribe Cymbarieae was a clade sister to the other parasitic lineages. *Cymbaria mongolica* and *C. daurica* were grouped into the monophyletic genus *Cymbaria*, which comprised a clade sister to the *Schwalbea*–*Siphonostegia* clade. Divergence time analyses (Figure 8; Table S5) revealed that the parasites diverged from autotrophic plants in the mid‐Eocene (42.95 Mya). The emergence of Cymbarieae predated the mid‐Oligocene (31.44 Mya) and the diversification of *Cymbaria* was estimated to occur around the late Miocene (6.72 Mya).

## 4 DISCUSSION

### 4.1 Pseudogenization/loss events of *ndh* genes and the unique *rbc*L– *mat*K inversion

It is acknowledged that the lifestyle transition from autotrophy to heterotrophy triggers the degradation of chloroplast genomes (Wicke & Naumann, 2018). Contrasting with the hypervariability of holoparasites, *Cymbaria* species and other Orobanchaceae hemiparasites exhibit high similarity to autotrophs in length, GC content, and intact genes. It has been confirmed that holoparasites are characterized by chloroplast genome reduction (Wicke et al., 2013). The high variability of holoparasites is explained by increases in pseudogenization and gene loss. However, patterns of variation in hemiparasites are diverse. The chloroplast genomes of hemiparasites in the family Orobanchaceae were more similar to those of autotrophs, which contrasts with the reduction in the genome size of hemiparasites in the order Santalales (Guo et al., 2021; Li et al., 2017; Shin & Lee, 2018). This might be attributed in part to GC‐biased gene conversion and mutational biases, which suggest that sophisticated mechanisms contribute to the stability (Niu et al., 2017). Angiosperms typically possess 113 plastid genes, consisting of 79 functional PCGs, 30 tRNA, and four rRNA genes (Wicke et al., 2011). Within the Orobanchaceae family, pseudogenes and gene losses were largely absent in autotrophs, occasionally observed in most hemiparasites, and common in nearly all holoparasites. This can be explained by the tendency for the chloroplast genomes of parasites to be reduced in size (Naumann et al., 2016; Wicke & Naumann, 2018). The chloroplast nicotinamide adenine dinucleotide phosphate (NAD(P)H)–dehydrogenase complex comprises 11 *ndh* genes (Ma et al., 2021), and the pseudogenization or loss of these genes represents the initial stage of reductive evolution. We have found that that *C. mongolica* has lost genes (*ndh*I, *ndh*J) and that *C. daurica* contains both pseudogenes (*ndh*F, *ndh*H) and lost genes (*ndh*A, *ndh*C, *ndh*E, *ndh*G, *ndh*I), indicating that these two species are in the initial phase of the autotroph‐to‐heterotroph transition. A previous study has confirmed that a hemiparasitic lifestyle can lead to an increase in the pseudogenization/loss of *ndh* genes (Li et al., 2021). This can be explained to some extent by the facultative root hemiparasitic lifestyle of the two *Cymbaria* species. The degradation of *ndh* genes affects several morphological and physiological traits and enhances the adaptation of plants to environmental stress (Sabater, 2021). The only inversion observed was that of *rbc*L–*mat*K in the LSC region, which most likely stems from a palindromic repeat‐mediated rearrangement. Inversions of the LSC fragments have also been observed in *Schwalbea americana* (Wicke et al., 2013) and *Siphonostegia chinensis* (Jiang et al., 2022); this distinct evolutionary mechanism among Orobanchaceae members might explain the unique phylogenetic position of the tribe Cymbarieae. This inversion has also been observed in *Codonopsis pilosula* subsp. *tangshen* (Yue et al., 2022) and *Avena sativa* (Liu et al., 2020). The codon usage bias of the two *Cymbaria* species might be affected by natural selection and mutation, as it has been observed in several other angiosperms (Lu et al., 2020). The number of repeats plays a key role in maintaining the stability of several angiosperms' chloroplast genome (Jansen et al., 2010). The chloroplast genome of *C. daurica* has more repetitive sequences than *C. mongolica*, suggesting that the stability of the former might be higher than that of the latter. Our findings suggest that A/T mononucleotide SSRs are dominant, and this is consistent with the high prevalence of AT richness (Liu et al., 2018).

### 4.2 Specific DNA barcodes for distinguishing the Mongolian herb *C. daurica* from its adulterant *C. mongolica*

Traditional Mongolian medicine continues to receive much clinical attention because of its distinctive properties and herb resources (Xu, Zhao, et al., 2022); at present, its use is still very important among the Mongolian populations of eastern Asia (Gula, 2021; Pitschmann et al., 2013). *Cymbaria daurica* has been used to treat pruritus, psoriasis, fetotoxicity, impetigo, and diabetes. The incidence of adulteration of *C. daurica* with its sister species *C. mongolica* is increasing, and this poses a threat to the clinical efficacy of the herb. The high similarity in morphology between *C. daurica* and *C. mongolica* is the root cause of this problem. Specifically, *C. daurica* has densely white sericeous anther locules that are 4–4.5 mm and apically pilose, whereas *C. mongolica* has pilose anther locules that are 3–3.6 mm and glabrous apically or occasionally with few hairs (Hong et al., 1998); thus, distinguishing between these two species is a major challenge in the non‐flowering stage. Distinguishing between these two *Cymbaria* species is exceedingly difficult because they only differ in anther morphology (Zhang et al., 2013). Some previous studies have suggested that specific barcodes are superior in performance to universal barcodes for distinguishing between morphologically similar species (Fang et al., 2023; Lu et al., 2022). Therefore, several divergence hotspot regions were first identified through sequence divergence and nucleotide variability, Then, we developed and validated specific DNA barcodes to distinguish between these two species. Overall, these four pairs of specific DNA barcodes could be used to accurately and rapidly identify which of the two species (*C. daurica* or *C. mongolica*) is especially important, given that the present sample is without the need to evaluate the morphological characteristics of the anther during flowering by specialized personnel.

### 4.3 Climate aridification and increasing host dependence accelerate the diversification of *Cymbaria*

Traditional Orobanchaceae has been merged with all hemiparasitic genera as well as a few holoparasitic genera formerly placed in Scrophulariaceae (Bennett & Mathews, 2006; dePamphilis et al., 1997; McNeal et al., 2013; Wolfe et al., 2005; Young et al., 1999) and the three autotrophic genera previously considered as sister taxa to Orobanchaceae (Schneeweiss, 2013; Xia et al., 2021). Moreover, the two new tribes Brandisieae and Pterygielleae have been proposed (Jiang et al., 2022; Yu et al., 2018). To date, Orobanchaceae includes nine well‐supported clades corresponding to nine tribes, i.e., the two autotrophic tribes Rehmannieae and Lindenbergieae, and the seven parasitic tribes Cymbarieae, Buchnereae, Orobancheae, Brandisieae, Pedicularideae, Rhinantheae, and Pterygielleae. The topology of the major clades and autotroph–parasite sister relationships revealed by our phylogenetic analyses are generally consistent with previous findings.

The hemiparasitic tribe Cymbarieae is distinguished from other tribes in the family Orobanchaceae by the presence of bracteoles, a tubular calyx that is weakly dorsiventral, a conspicuously two‐lipped corolla, and anthers with two mostly rounded and equal thecae (Fischer, 2004). Cymbarieae has traditionally been considered sister to all other parasitic lineages (Bennett & Mathews, 2006; McNeal et al., 2013). However, a recent study has challenged this classification after reporting that the holoparasitic tribe Orobancheae is sister to all other parasitic members (Li et al., 2019). Our findings supported the traditional classification of the Cymbarieae, in contrast to the results of Li et al. (2019) who used only a few nuclear genes. Such inconsistency of cytoplasmic‐nuclear conflict might be explained by incomplete lineage sorting or deep coalescence (Maddison, 1997). Resolving these early nodes will require a coalescent approach that involves many genes with different histories. In addition, the Cymbarieae was found to be sister to the remaining parasitic lineages, and the hemiparasites evolved earlier than the holoparasites, which is consistent with the progressive nature of the evolution of increased host dependence (Xu, Zhang, et al., 2022).

Within parasitic plants, the hemiparasitic tribe Cymbarieae diverged from its closest lineages in the mid‐Oligocene (31.44 Mya), which was most likely induced by global climate cooling and the retreat of the Tethys Sea during the Eocene–Oligocene Transition at 34 Mya (Abels et al., 2011). The emergence and further diversification of hemiparasites might be attributed to the rapid expansion of grasslands during the Oligocene (Torsvik & Cocks, 2016), which would have provided them with opportunities to exploit host plants. The two *Cymbaria* species diversified in the late Miocene (6.72 Mya), which was driven by the final uplift of the Qinghai–Tibetan Plateau, the onset of the East Asian monsoon, and the large accumulation of dust in the Loess Plateau from 10 to 7 Mya (Favre et al., 2015; Zhisheng et al., 2001, 2015). Both climate aridification and the increase in host steppe vegetation (Hurka et al., 2019) likely accelerated the adaptive evolution of *Cymbaria* species in the steppe region.

## 5 CONCLUSIONS

We characterized the chloroplast genomes of two *Cymbaria* species and conducted a comparative analysis using chloroplast genomes across 54 Orobanchaceae species. The chloroplast genomes of *C. mongolica* and *C. daurica* have a typical quadripartite structure, and their total lengths are 149,431 bp and 151,545 bp, respectively. Although the chloroplast genomes of holoparasites are hypervariable, those of *Cymbaria* species, other hemiparasites, and autotrophs are highly similar regarding genome size, GC content, and intact genes. The pseudogenization/loss of *ndh* genes might be associated with the facultative root hemiparasitic habits of *Cymbaria*. The *rbc*L–*mat*K inversion in the LSC region most likely stemmed from a palindromic repeat‐mediated rearrangement. Specific DNA barcodes were developed using four pairs of primers (*CymN1*, *CymN2*, *CymY*, and *CymR*) that amplified sequences from the divergent hotspot regions to distinguish the traditional Mongolian herb *C. daurica* from its adulterant *C. mongolica*. The genus *Cymbaria* and the *Schwalbea*–*Siphonostegia* clade were clustered in the tribe Cymbarieae. This tribe comprised an independent clade sister to the remaining parasitic lineages, which does not match with the relationships hypothesized in a recent study. The monophyletic genus *Cymbaria* diversified during the late Miocene period (6.72 Mya). The aridification of the climate and the expansion of host steppe vegetation likely promoted the adaptive evolution of *Cymbaria* species in the Mongol–‐Chinese steppe region. Our results provide key information for clarifying the taxonomic identification, phylogenetic placement, and reductive evolution of *Cymbaria*; our findings will also help assessments of the authenticity of the traditional Mongolian medicine “*Xinba*.”

## AUTHOR CONTRIBUTIONS

**Yang Ma:** Conceptualization (equal); investigation (lead); methodology (lead); resources (lead); software (lead); validation (lead); visualization (lead); writing – original draft (lead); writing – review and editing (lead). **Jordi López‐Pujol:** Writing – review and editing (supporting). **Dongqing Yan:** Investigation (supporting); resources (supporting). **Zhen Zhou:** Investigation (supporting); resources (supporting). **Zekun Deng:** Investigation (supporting); resources (supporting). **Jianming Niu:** Conceptualization (equal); funding acquisition (lead); project administration (lead); supervision (lead).

## FUNDING INFORMATION

Major Science and Technology Projects of Inner Mongolia Autonomous Region, Grant/Award Number: 2019ZD008; National Natural Science Foundation of China, Grant/Award Number: 31860106 and 322603042.

## CONFLICT OF INTEREST STATEMENT

The authors declare that they have no known competing financial interests or personal relationships that could have appeared to influence the work reported in this paper.

## Supporting information


Appendix S1

## Data Availability

The data used in this study are openly available in the National Center for Biotechnology Information (NCBI) database (https://www.ncbi.nlm.nih.gov) with GenBank accession numbers NC_064388.1 (*Cymbaria mongolica*) and NC_064104.1 (*Cymbaria daurica*).
