# Supplementary material for: Complete chloroplast genomes of the hemiparasitic genus Cymbaria: Insights into comparative analysis, development of molecular markers, and phylogenetic relationships
Source: Ecol Evol. 2024 Jul 3;14(7):e11677. doi: 10.1002/ece3.11677 (PMC11221886; doi:10.1002/ece3.11677)
Supplement: Supplementary file 1 — Appendix S1 [file ECE3-14-e11677-s001.docx]

**APPENDIX**


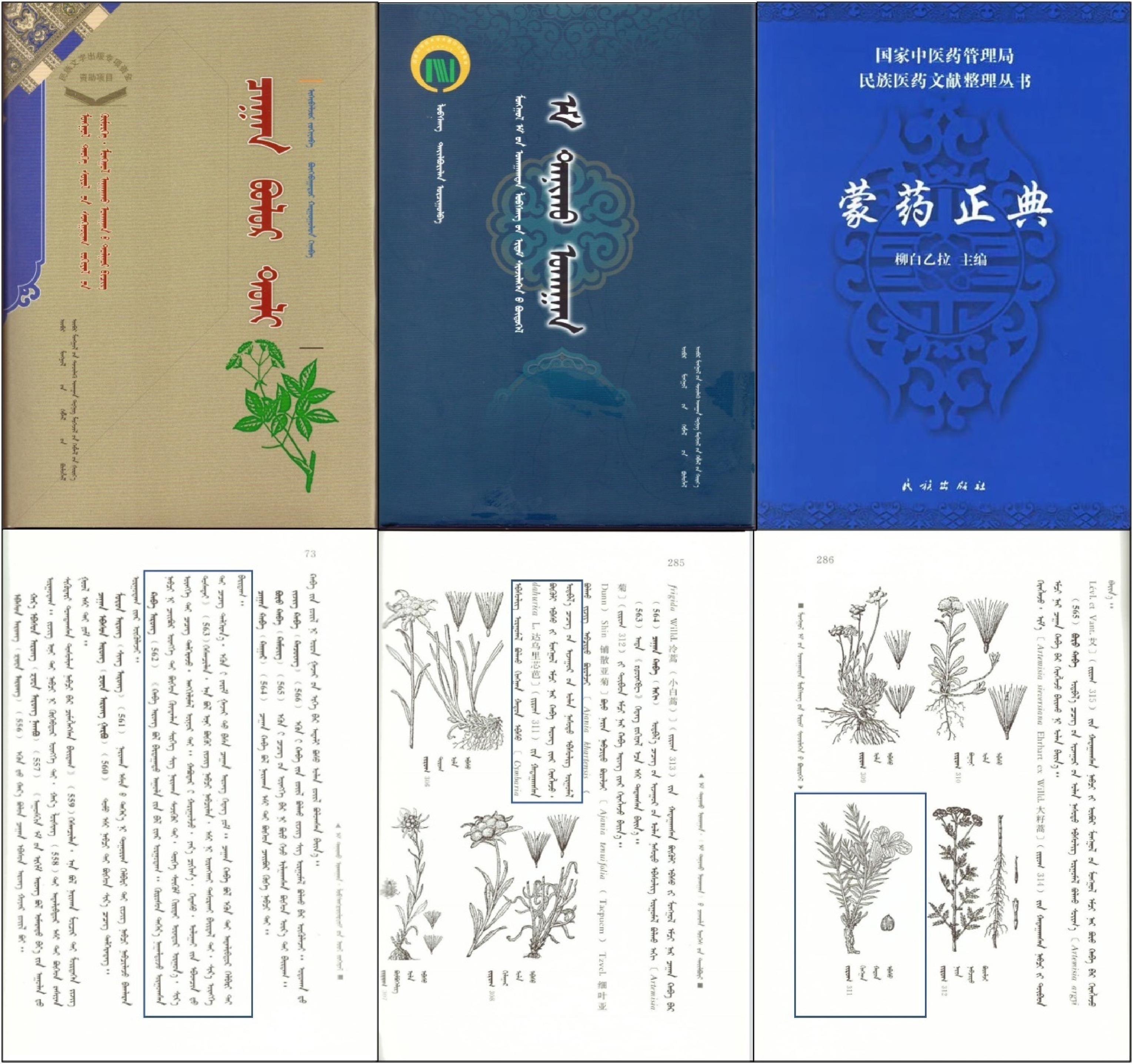


**Figure S1.** Mongolian pharmaceutical classic literature describing the herb C. daurica (Huang et al., 2023).


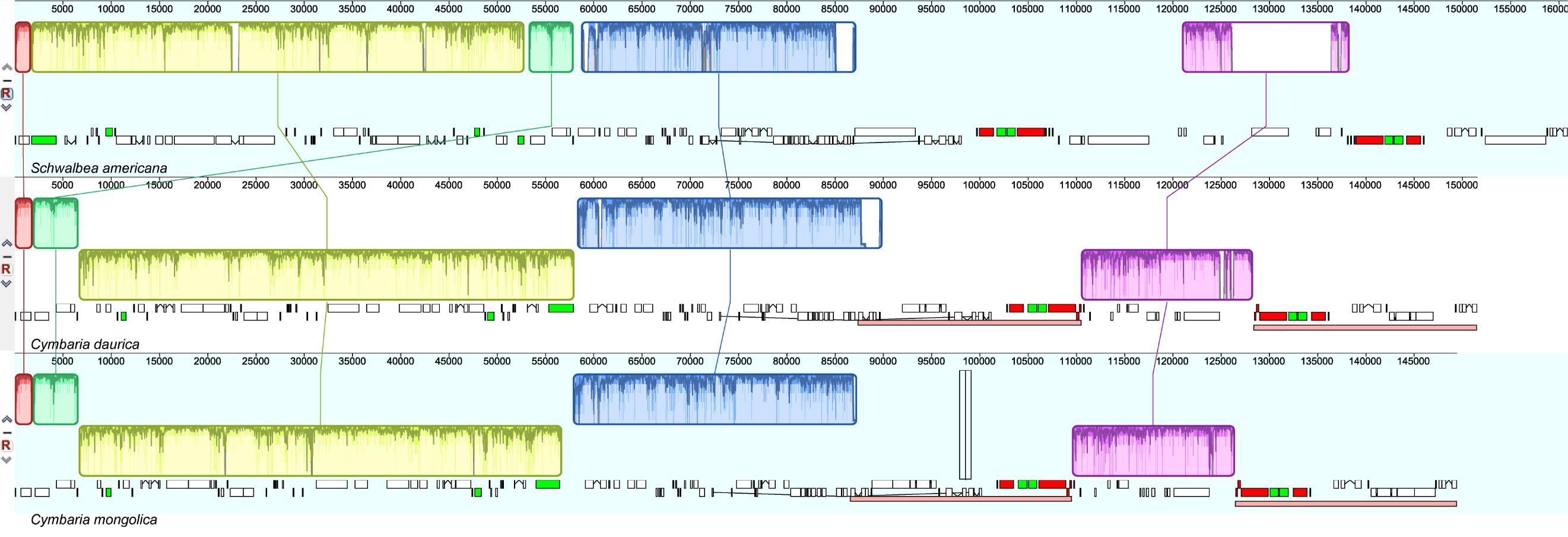


**Figure S2.** Collinearity of two *Cymbaria* chloroplast genomes using Mauve program with *Schwalbea americana* as the reference.


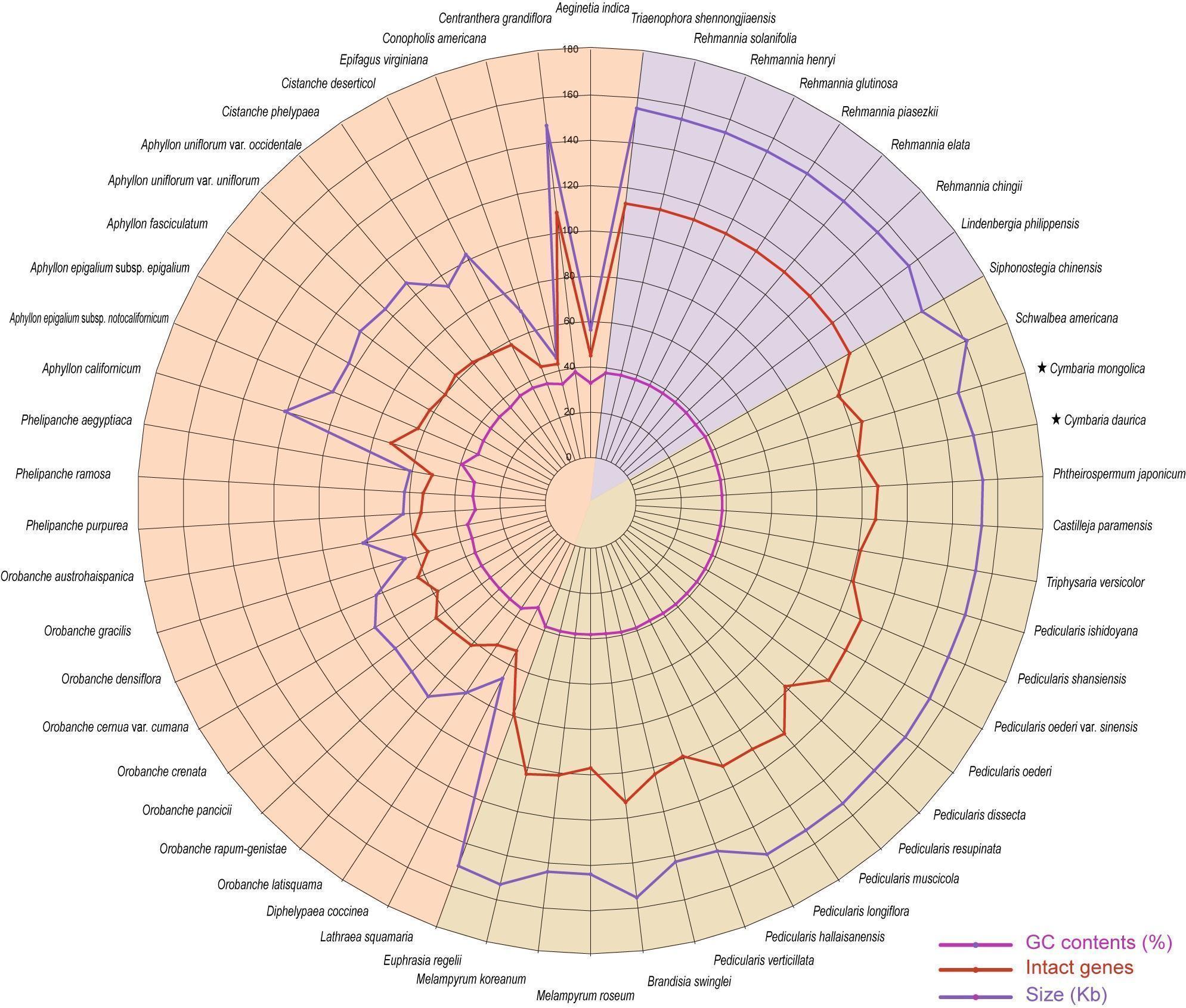


**Figure S3.** Radar chart of complete chloroplast genome comparison of 54 Orobanchaceae species. From outside to inside: genome size, intact genes, and GC content. Background colors of purple, brown, and orange represent autotroph, hemiparasite, and holoparasite species, respectively.


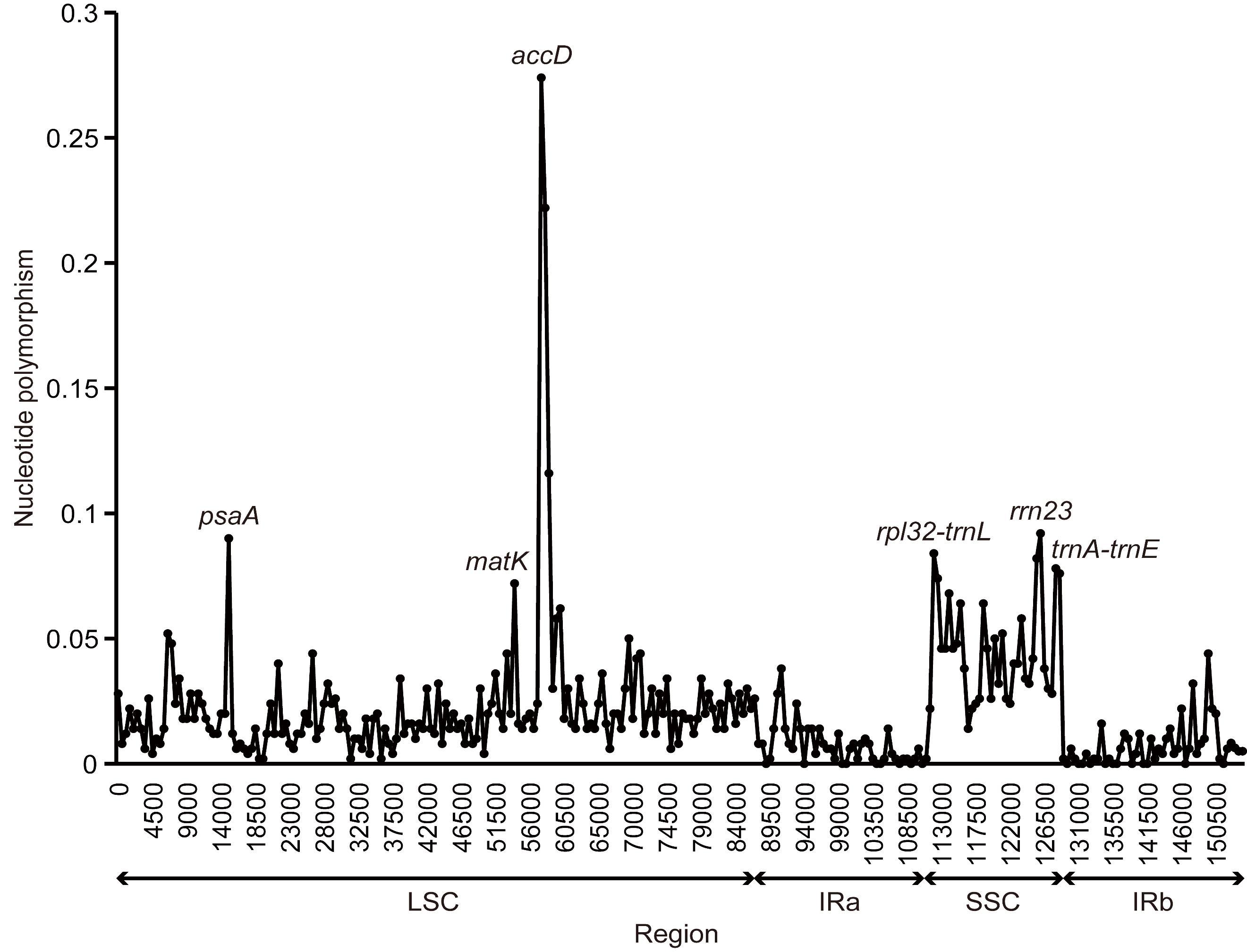


**Figure** **S4.** Sliding window analysis of two *Cymbaria* chloroplast genomes.


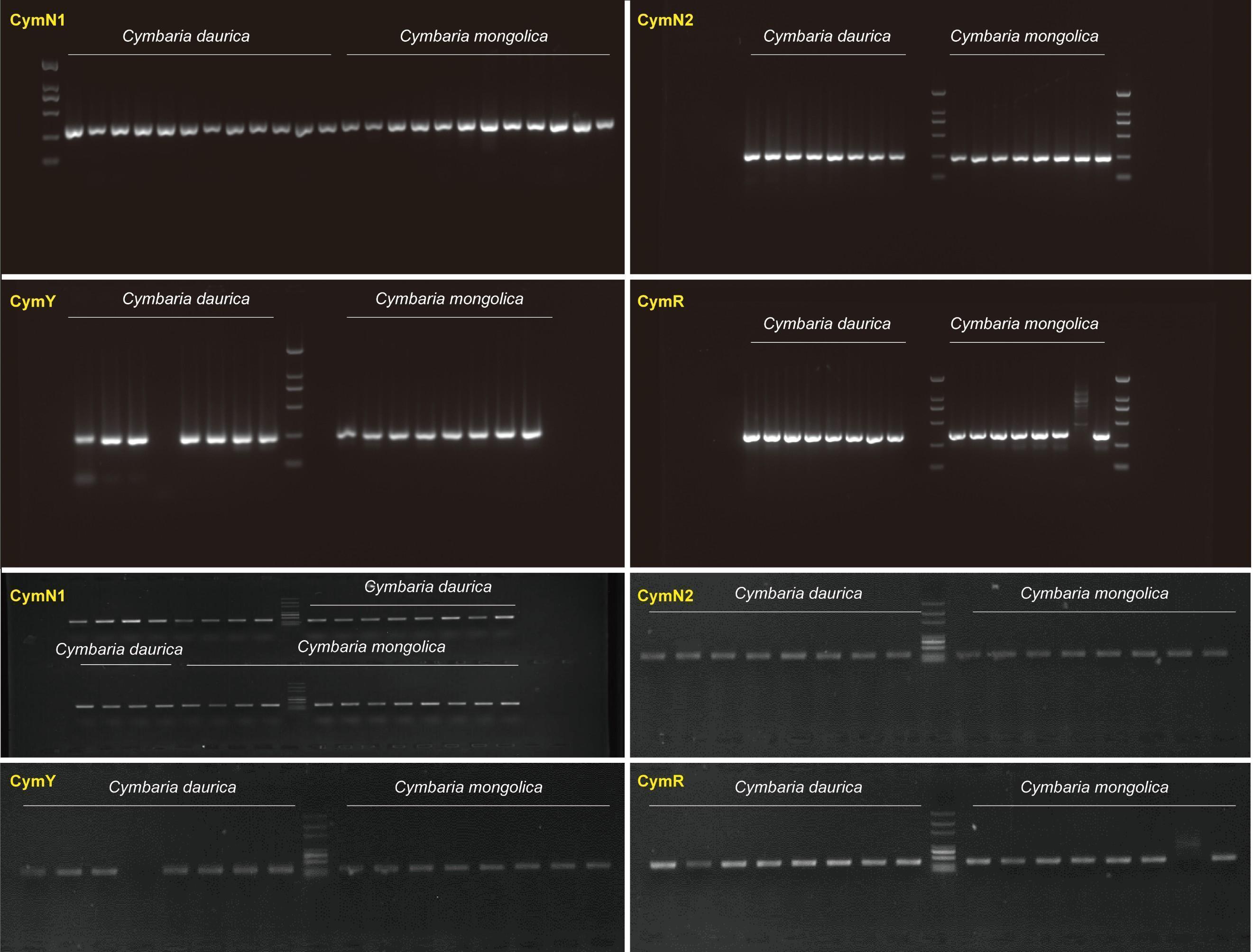


**Figure S5.** The original and uncropped agarose gel electrophoresis of the amplification of DNA barcodes

**
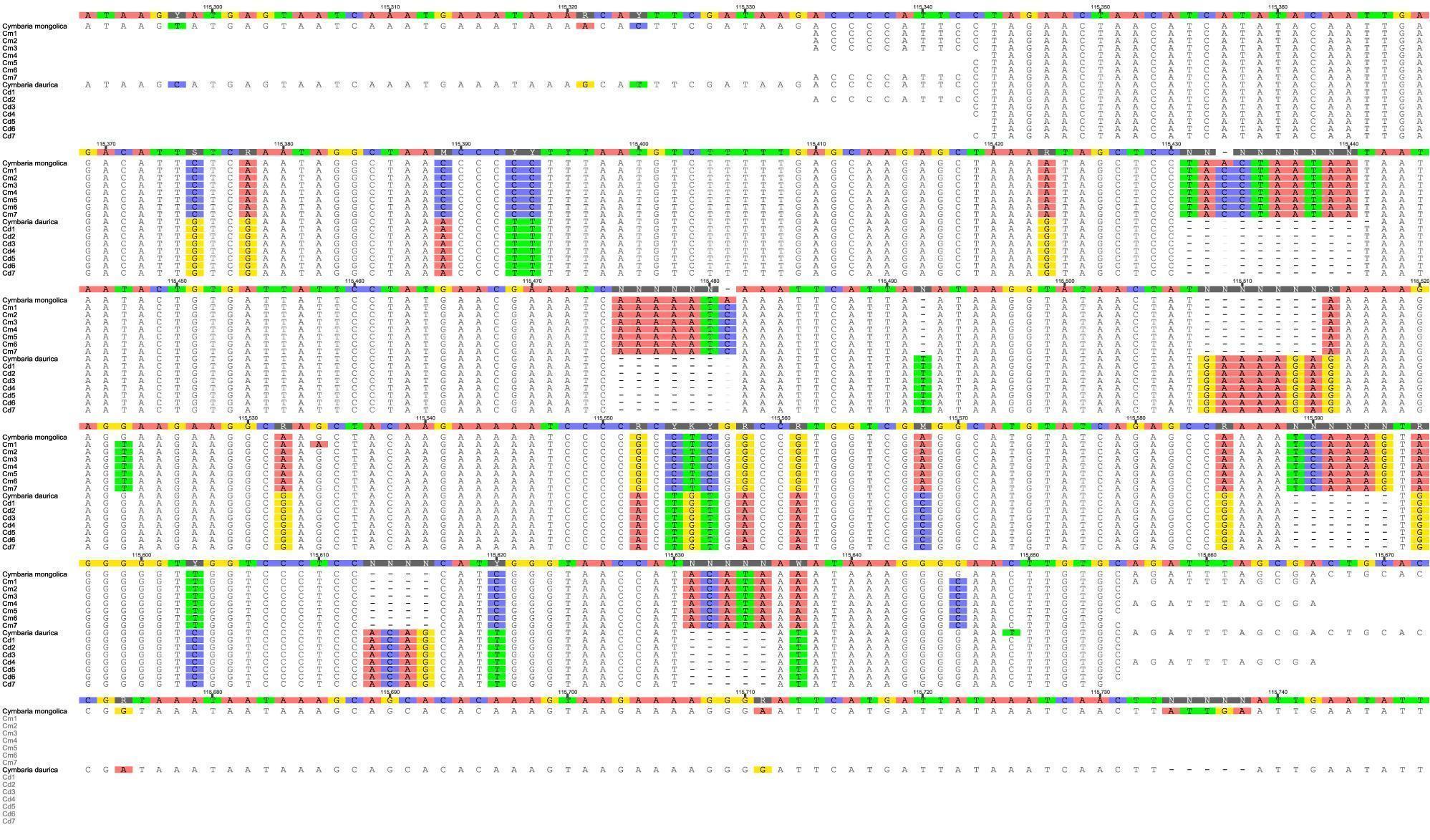
**

**Figure S6.** Sequence alignment of the 14 amplified samples based on CymN1 maker, using two *Cymbaria* chloroplast genomes as references.

**
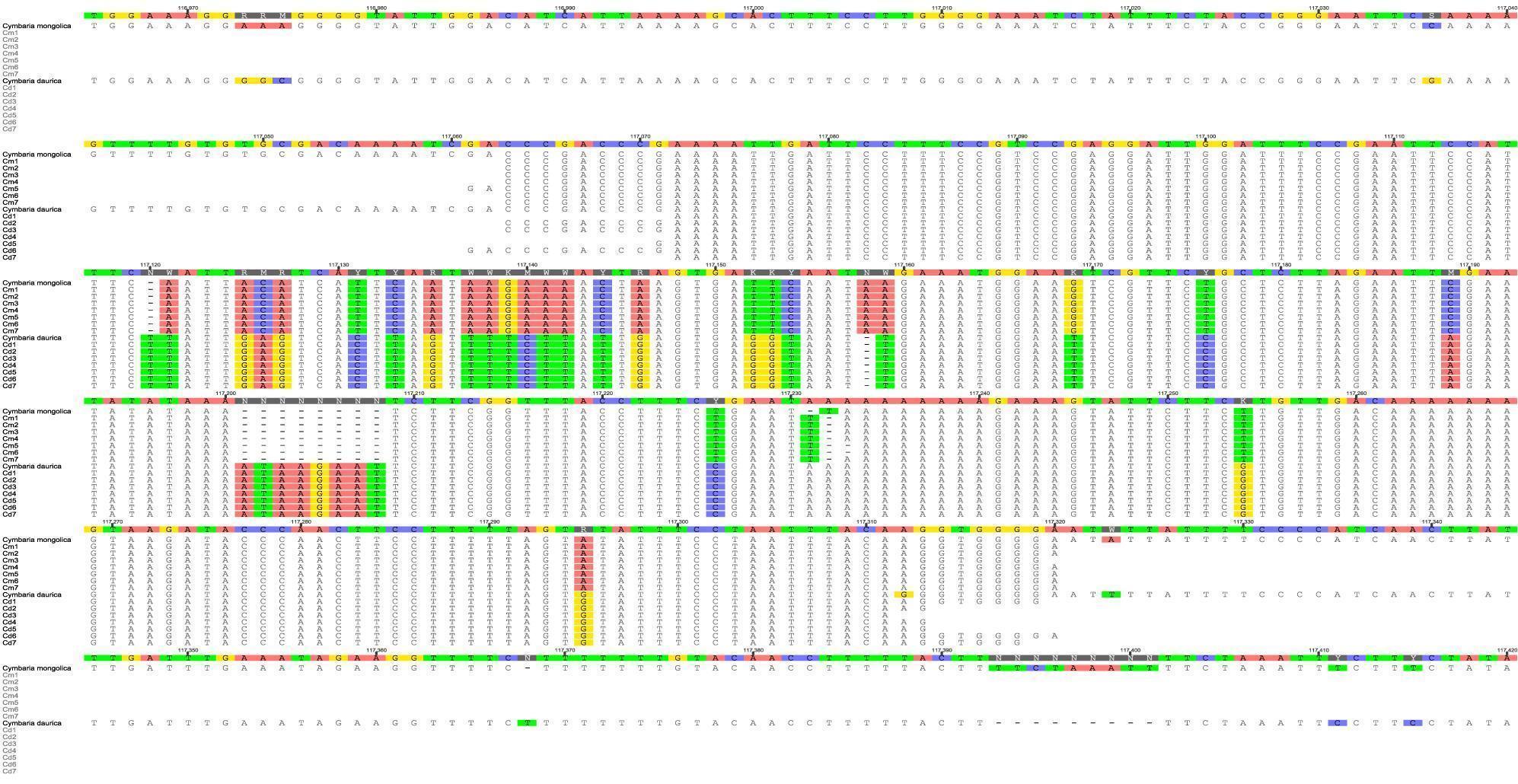
**

**Figure S7.** Sequence alignment of the 14 amplified samples based on CymN2 marker, using two *Cymbaria* chloroplast genomes as references.

**
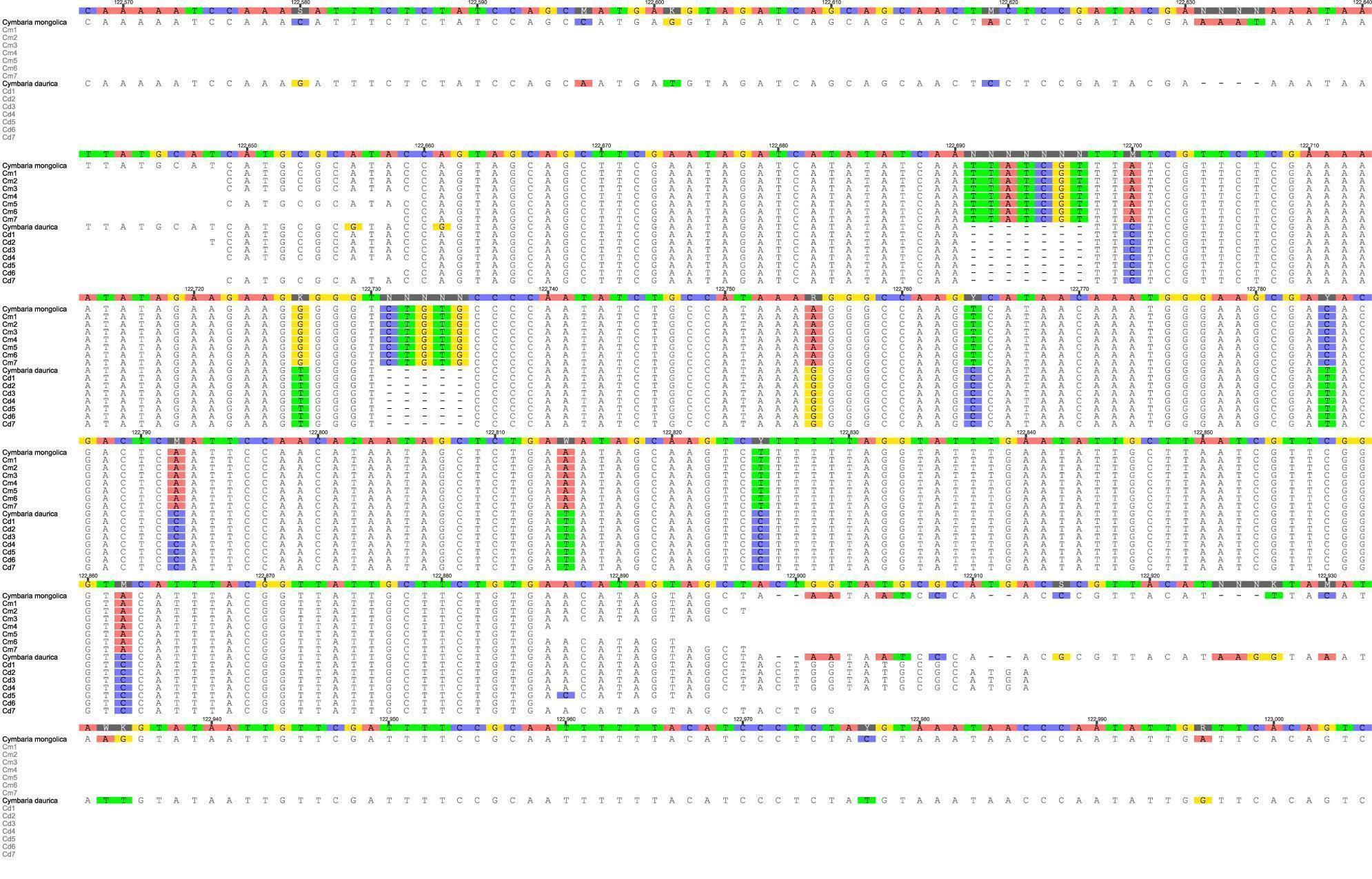
**

**Figure S8.** Sequence alignment of the 14 amplified samples based on CymY marker, using two *Cymbaria* chloroplast genomes as references.

**
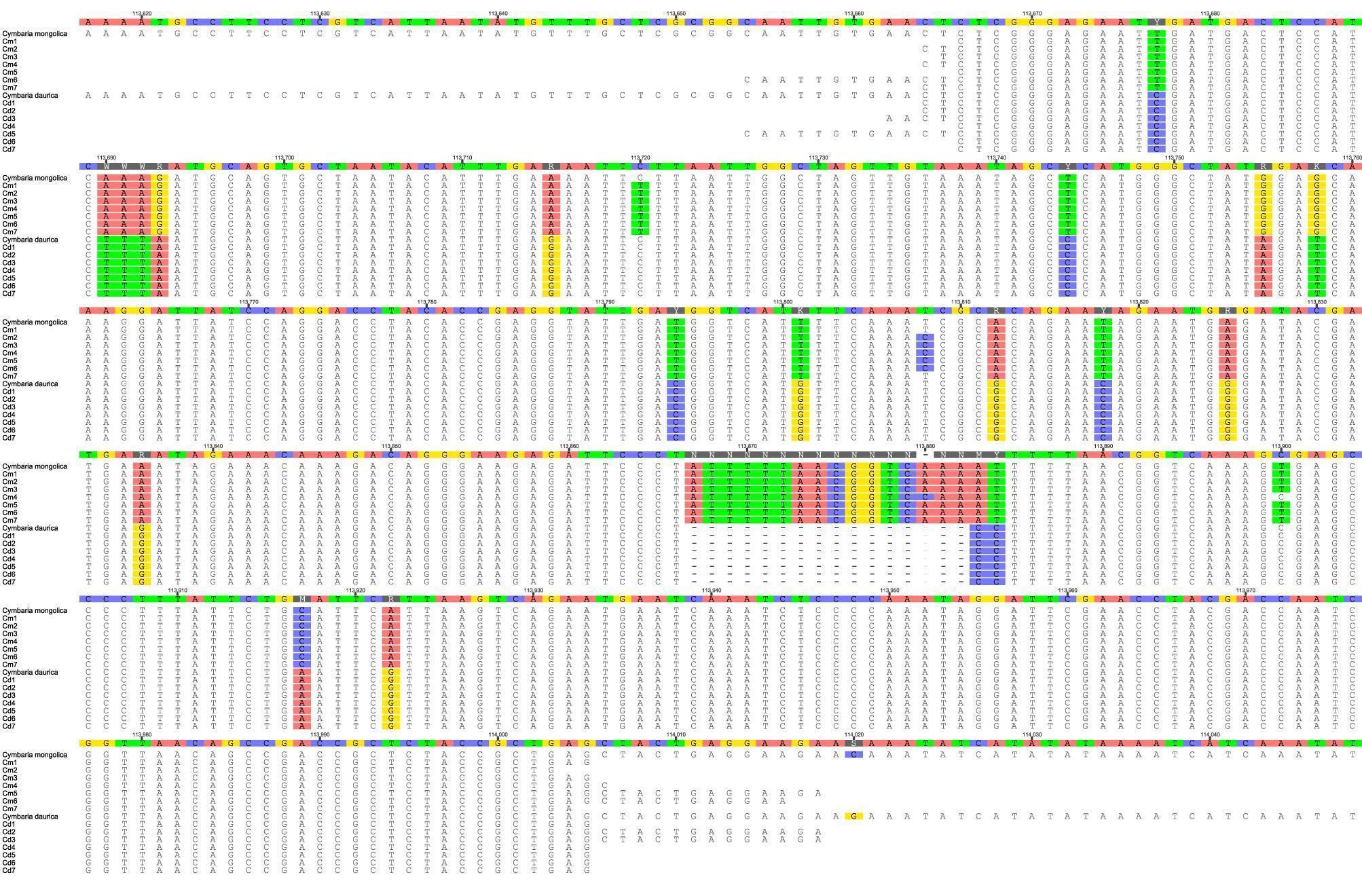
**

**Figure S9.** Sequence alignment of the 14 amplified samples based on CymR marker, using two *Cymbaria* chloroplast genomes as references.

**
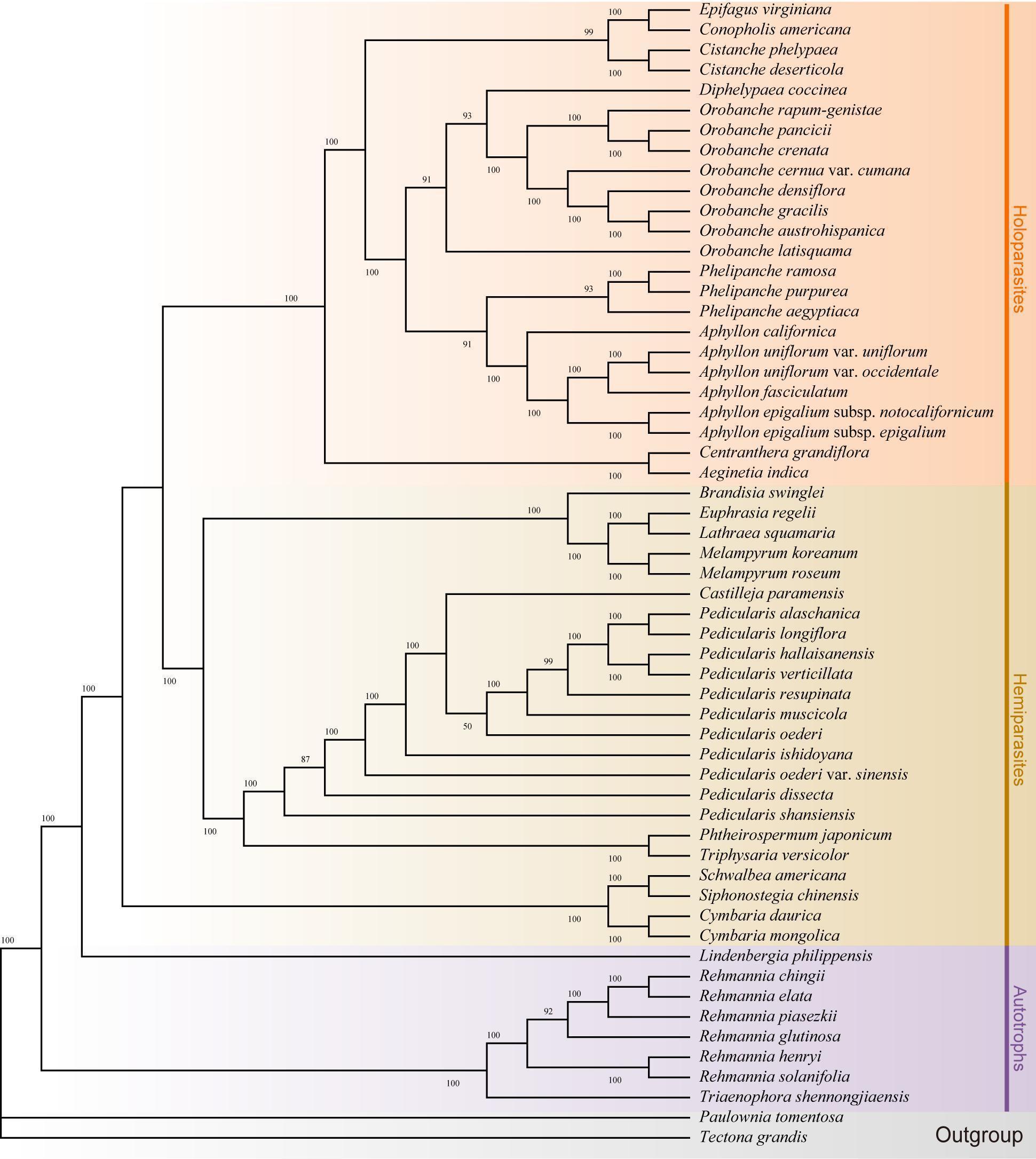
**

**Figure S10.** Phylogenetic relationship inferred from ML based on complete chloroplast genome sequences of 54 Orobanchaceae species.

**
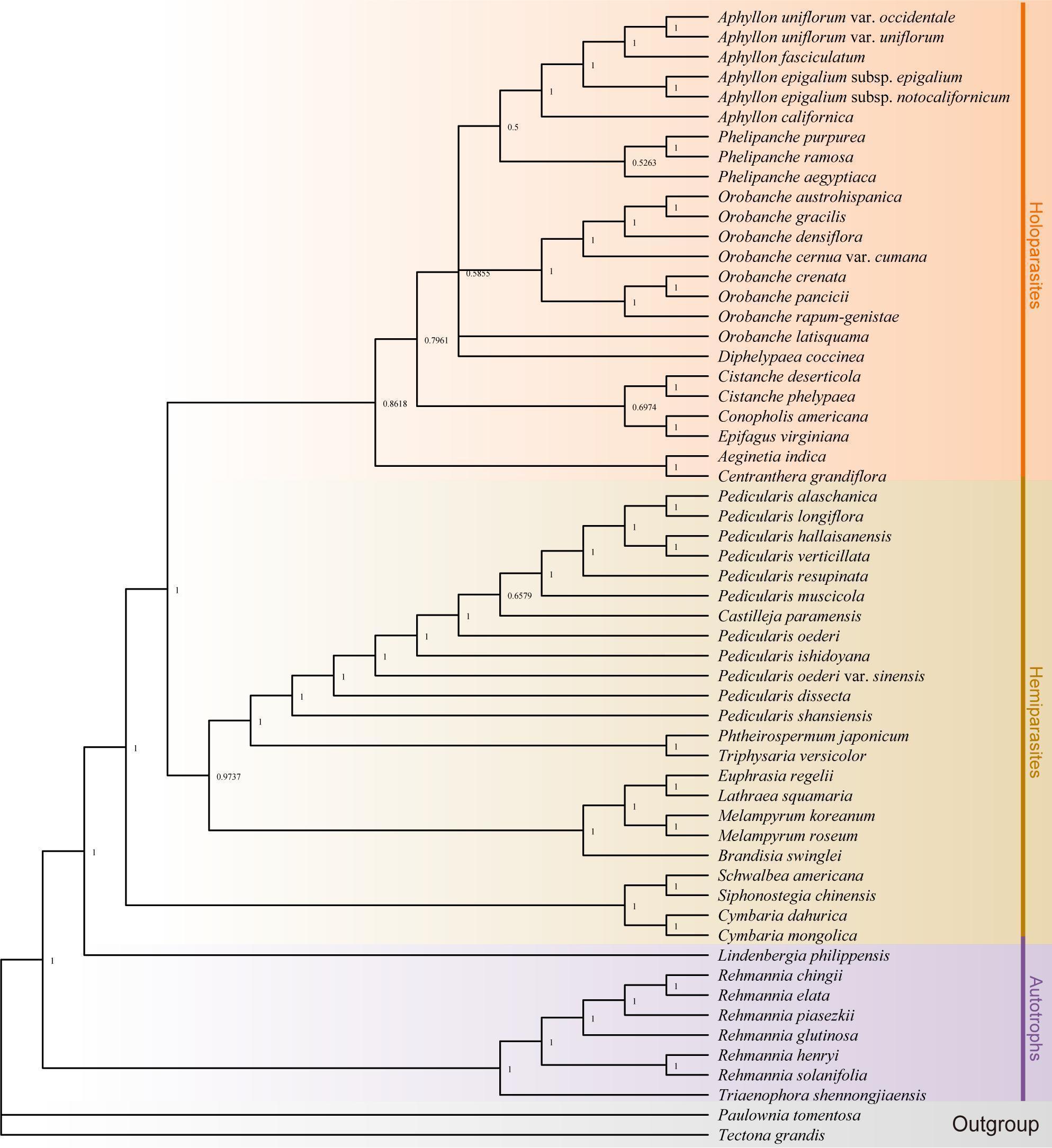
**

**Figure S11.** Phylogenetic relationship inferred from BI based on complete chloroplast genome sequences of 54 Orobanchaceae species.


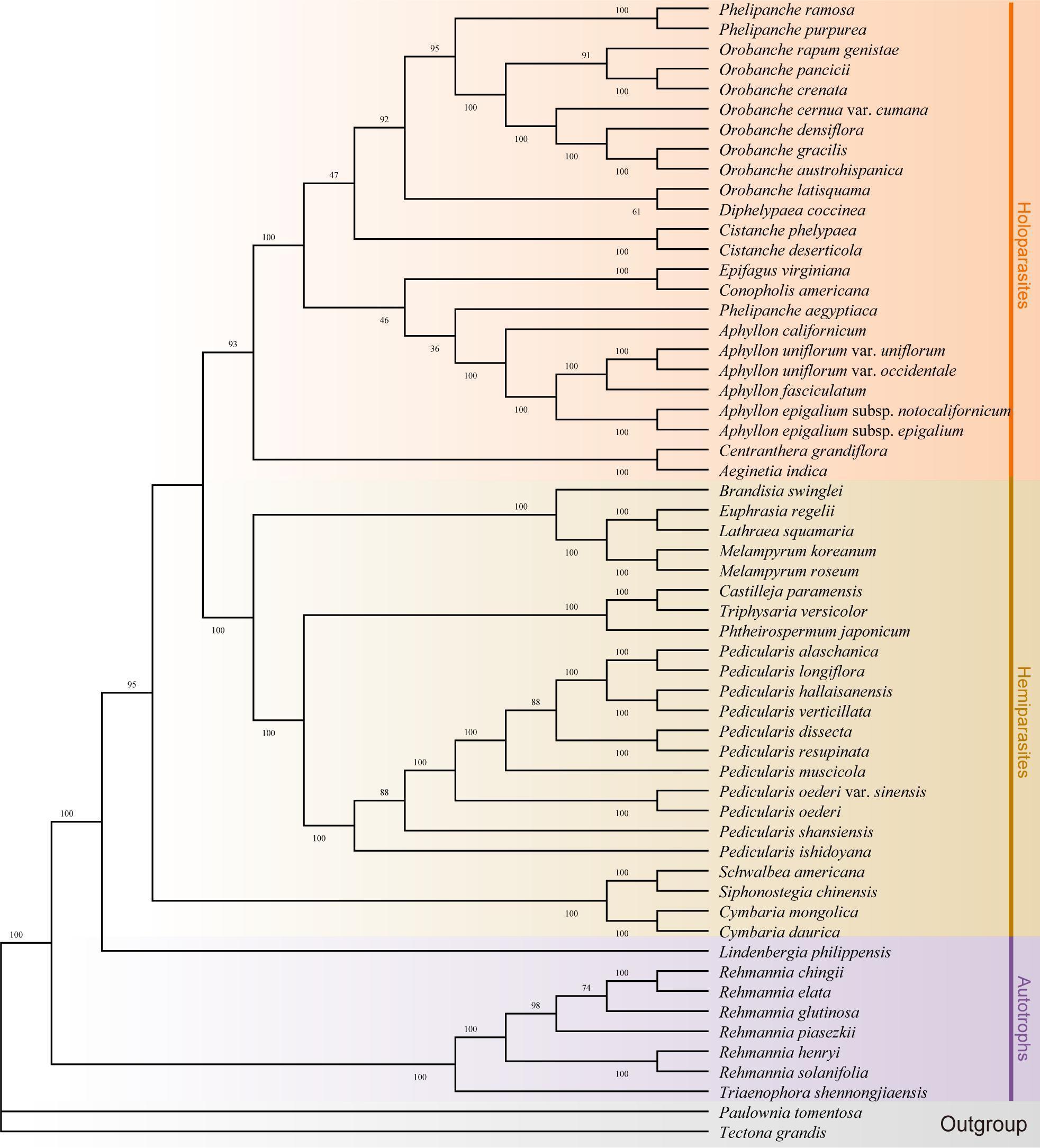


**Figure S12.** Phylogenetic relationship inferred from ML based on coding DNA sequences of 54 Orobanchaceae species.


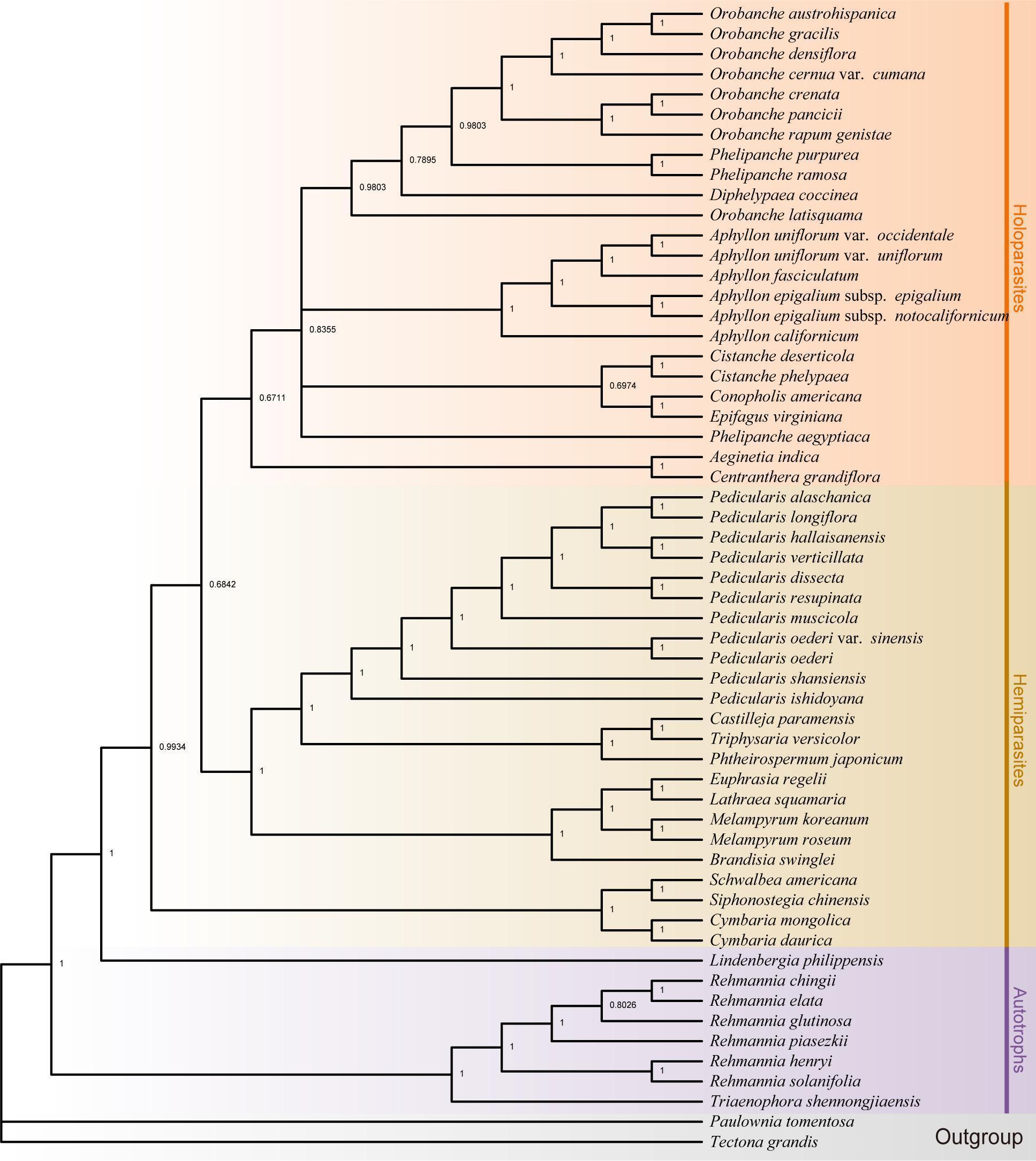


**Figure S13.** Phylogenetic relationship inferred from BI based on coding DNA sequences of 54 Orobanchaceae species.

**Table S1.** Gene contents in two *Cymbaria* chloroplast genomes.

| Category | Gene group | Gene name | |
| --- | --- | --- | --- |
|  |  | *C. mongolica* | *C. daurica* |
| Photosynthesis | Subunits of photosystem I | *psa*A, *psa*B, *psa*C, *psa*I, *psa*J | |
|  | Subunits of photosystem II | *psb*A, *psb*B, *psb*C, *psb*D, *psb*E, *psb*F, *psb*H, *psb*I, *psb*J, *psb*K, *psb*L, *psb*M, *psb*N, *psb*T, *psb*Z | |
|  | Subunits of NADH dehydrogenase | *ndh*A, *ndh*B^a^, *ndh*C, *ndh*D, *ndh*E, *ndh*F, *ndh*G, *ndh*H, *ndh*K | *ndh*F^Ψ^, *ndh*H^Ψ^, *ndh*B^a^, *ndh*D, *ndh*F, *ndh*H, *ndh*J, *ndh*K |
|  | Subunits of cytochrome b/f complex | *pet*A, *pet*B^a^, *pet*D, *pet*G, *pet*L, *pet*N | |
|  | Subunits of ATP synthase | *atp*A, *atp*B, *atp*E, *atp*F^a^, *atp*H, *atp*I | |
|  | Large subunit of rubisco | *rbc*L | |
|  | Subunits of photochlorophyllide reductase | - | |
| Self-replication | Proteins of large ribosomal subunit | *rpl*14, *rpl*16, *rpl*2^a^, *rpl*20, *rpl*22, *rpl*23, *rpl*32, *rpl*33, *rpl*36 | |
|  | Proteins of small ribosomal subunit | *rps*11, *rps*12^b^, *rps*14, *rps*15, *rps*16^a^, *rps*18, *rps*2, *rps*3^a^, *rps*4, *rps*7(2), *rps*8 | |
|  | Subunits of RNA polymerase | *rpo*A, *rpo*B, *rpo*C1, *rpo*C2 | |
|  | Ribosomal RNAs | *rrn*16S, *rrn*23S, *rrn*4.5S, *rrn*5S | |
|  | Transfer RNAs | *trn*A-UGC^a^, *trn*C-GCA, *trn*D-GUC, *trn*E-UUC^a^, *trn*F-GAA, *trn*G-GCC, *trn*H-GUG, *trn*K-UUU^a^, *trn*L-CAA, *trn*L-UAA^a^, *trn*L-UAG, *trn*M-CAU, *trn*N-GUU, *trn*P-UGG, *trn*Q-UUG, *trn*R-ACG, *trn*R-UCU, *trn*S-CGA^a^, *trn*S-GCU, *trn*S-GGA, *trn*S-UGA, *trn*T-GGU, *trn*T-UGU, *trn*V-GAC, *trn*W-CCA, *trn*Y-GUA | |
| Other genes | Maturase | *mat*K | |
|  | Protease | *clp*P | |
|  | Envelope membrane protein | *cem*A | |
|  | Acetyl-CoA carboxylase | *acc*D^a^ | |
|  | c-type cytochrome synthesis gene | *ccs*A | |
|  | Translation initiation factor | *inf*A | |
|  | Other | - | |
| Genes of unknown function | Conserved hypothetical chloroplast ORF | *ycf*1^Ψ^, *ycf*2^b^, *ycf*3^b^, *ycf*4 | |

Notes: Gene^a^: containing one intron; Gene^b^: containing two introns; Gene^Ψ^: pseudogenes

**Table S2.** Characteristics of 54 Orobanchaceae chloroplast genomes.

| Accession | Lifestlye | Species | Size (kbp) | GC content (%) | Intact genes | Protein-coding genes | tRNA genes | rRNA genes |
| --- | --- | --- | --- | --- | --- | --- | --- | --- |
| MW851293 | Holoparasites | *Aeginetia_indica* | 56.381 | 32.9 | 45 | 27 | 17 | 1 |
| MW262988 | Holoparasites | *Centranthera_grandiflora* | 147.655 | 38.2 | 109 | 75 | 30 | 4 |
| NC_023131 | Holoparasites | *Conopholis_americana* | 45.673 | 33.9 | 43 | 24 | 19 | 0 |
| NC_001568 | Holoparasites | *Epifagus_virginiana* | 70.028 | 36 | 44 | 22 | 22 | 0 |
| NC_021111 | Holoparasites | *Cistanche_deserticola* | 102.657 | 36.8 | 58 | 26 | 28 | 4 |
| NC_025642 | Holoparasites | *Cistanche_phelypaea* | 94.380 | 36.6 | 59 | 27 | 29 | 3 |
| MH499250 | Holoparasites | *Aphyllon_uniflorum_*var._*occidentale* | 106.661 | 35.1 | 61 | 29 | 29 | 3 |
| MH580290 | Holoparasites | *Aphyllon_uniflorum_*var._*uniflorum* | 104.640 | 35.3 | 62 | 30 | 29 | 3 |
| NC_039679 | Holoparasites | *Aphyllon_fasciculatum* | 106.796 | 35 | 60 | 28 | 29 | 3 |
| MH050785 | Holoparasites | *Aphyllon_epigalium_*subsp._*epigalium* | 56.381 | 32.9 | 45 | 13 | 30 | 2 |
| MH050786 | Holoparasites | *Aphyllon_epigalium_*subsp.*_notocalifornicum* | 103.932 | 34.1 | 63 | 30 | 30 | 3 |
| NC_025651 | Holoparasites | *Aphyllon californicum* | 120.840 | 39.3 | 72 | 38 | 30 | 4 |
| KU212370 | Holoparasites | *Phelipanche_aegyptiaca* | 60.947 | 32.1 | 51 | 27 | 22 | 2 |
| NC_023465 | Holoparasites | *Phelipanche_ramosa* | 62.304 | 32.1 | 54 | 28 | 24 | 2 |
| NC_023132 | Holoparasites | *Phelipanche_purpurea* | 62.891 | 31 | 55 | 29 | 24 | 2 |
| NC_031441 | Holoparasites | *Orobanche_austrohispanica* | 81.994 | 35.1 | 59 | 30 | 27 | 2 |
| NC_023464 | Holoparasites | *Orobanche_gracilis* | 65.533 | 34.6 | 55 | 26 | 26 | 3 |
| NC_031442 | Holoparasites | *Orobanche_densiflora* | 83.024 | 35.5 | 63 | 33 | 28 | 2 |
| KT387722 | Holoparasites | *Orobanche_cernua_*var.*_cumana* | 89.851 | 35.5 | 58 | 28 | 27 | 3 |
| NC_024845 | Holoparasites | *Orobanche_crenata* | 87.529 | 35.2 | 65 | 34 | 27 | 4 |
| NC_031443 | Holoparasites | *Orobanche_pancicii* | 88.525 | 35.2 | 63 | 33 | 27 | 3 |
| NC_031444 | Holoparasites | *Orobanche_rapum-genistae* | 91.517 | 35.4 | 62 | 32 | 26 | 4 |
| NC_025641 | Holoparasites | *Orobanche_latisquama* | 80.361 | 35.7 | 55 | 27 | 26 | 2 |
| NC_043877 | Holoparasites | *Diphelypaea_coccinea* | 66.616 | 31.7 | 53 | 26 | 24 | 3 |
| NC_027838 | Holoparasites | *Lathraea_squamaria* | 150.504 | 38.1 | 79 | 46 | 29 | 4 |
| NC_045041 | Hemiparasites | *Euphrasia_regelii* | 153.026 | 38.2 | 103 | 69 | 30 | 4 |
| NC_057523 | Hemiparasites | *Melampyrum_koreanum* | 143.865 | 38.2 | 101 | 67 | 30 | 4 |
| NC_053791 | Hemiparasites | *Melampyrum_roseum* | 143.896 | 38.1 | 97 | 67 | 26 | 4 |
| NC_042954 | Hemiparasites | *Brandisia_swinglei* | 155.344 | 38.1 | 113 | 79 | 30 | 4 |
| MT040753 | Hemiparasites | *Pedicularis_verticillata* | 142.733 | 38.6 | 103 | 69 | 30 | 4 |
| NC_037433 | Hemiparasites | *Pedicularis_hallaisanensis* | 143.469 | 38.7 | 99 | 65 | 30 | 4 |
| NC_046852 | Hemiparasites | *Pedicularis_longiflora* | 153.547 | 38.1 | 110 | 76 | 30 | 4 |
| NC_046853 | Hemiparasites | *Pedicularis muscicola* | 152.907 | 38.3 | 110 | 76 | 30 | 4 |
| NC_046397 | Hemiparasites | *Pedicularis_resupinata* | 153.145 | 38.4 | 113 | 79 | 30 | 4 |
| NC_056312 | Hemiparasites | *Pedicularis_dissecta* | 152.120 | 38.3 | 98 | 64 | 30 | 4 |
| NC_046854 | Hemiparasites | *Pedicularis_oederi* | 153.139 | 38.4 | 111 | 77 | 30 | 4 |
| MW770457 | Hemiparasites | *Pedicularis_oederi_*var.*_sinensis* | 152.770 | 38.3 | 110 | 76 | 30 | 4 |
| NC_058762 | Hemiparasites | *Pedicularis_shansiensis* | 151.902 | 38.3 | 110 | 78 | 28 | 4 |
| NC_029700 | Hemiparasites | *Pedicularis_ishidoyana* | 152.571 | 38.1 | 101 | 67 | 30 | 4 |
| NC_053793 | Hemiparasites | *Triphysaria_versicolor* | 152.583 | 38.2 | 101 | 73 | 24 | 4 |
| NC_031805 | Hemiparasites | *Castilleja_paramensis* | 152.926 | 38.2 | 106 | 72 | 30 | 4 |
| NC_053792 | Hemiparasites | *Phtheirospermum_japonicum* | 153.397 | 38.1 | 107 | 79 | 24 | 4 |
| NC_064388 | Hemiparasites | *Cymbaria_daurica* | 151.545 | 38.2 | 100 | 70 | 26 | 4 |
| NC_064104 | Hemiparasites | *Cymbaria mongolica* | 149.431 | 38 | 105 | 75 | 26 | 4 |
| NC_023115 | Hemiparasites | *Schwalbea_americana* | 160.910 | 38.1 | 99 | 73 | 24 | 2 |
| NC_046038 | Hemiparasites | *Siphonostegia_chinensis* | 148.961 | 38.4 | 112 | 78 | 30 | 4 |
| NC_022859 | Autotrophs | *Lindenbergia_philippensis* | 155.103 | 37.8 | 113 | 79 | 30 | 4 |
| NC_033534 | Autotrophs | *Rehmannia_chingii* | 154.055 | 38 | 113 | 79 | 30 | 4 |
| NC_034312 | Autotrophs | *Rehmannia_elata* | 153.772 | 38 | 113 | 79 | 30 | 4 |
| NC_034311 | Autotrophs | *Rehmannia_piasezkii* | 153.952 | 37.9 | 113 | 79 | 30 | 4 |
| NC_034308 | Autotrophs | *Rehmannia_glutinosa* | 153.622 | 38 | 113 | 79 | 30 | 4 |
| NC_034309 | Autotrophs | *Rehmannia_henryi* | 153.890 | 37.9 | 113 | 79 | 30 | 4 |
| NC_034310 | Autotrophs | *Rehmannia_solanifolia* | 153.989 | 37.9 | 113 | 79 | 30 | 4 |
| NC_039781 | Autotrophs | *Triaenophora_shennongjiaensis* | 155.319 | 37.7 | 113 | 79 | 30 | 4 |

Note: The statistics of gene numbers here refer to 113 unique plastid genes in the typical angiosperms. The duplicated genes are not included.

**Table S3.** Four pairs of primers for amplifying DNA barcodes.

| Name | Sequence (5’ to 3’) | Size (bp) | Position |
| --- | --- | --- | --- |
| CymN1_F | agaccccattcctagaactaaca | 23 | *ndhD-ndhG* |
| CymN1_R | agtcgctaaatctgcacaagt | 21 |  |
| CymN2_F | gacccgacccgaaaattgat | 20 | *ndhD-ndhG* |
| CymN2_R | tccccaccttgtaaattaggaaa | 23 |  |
| CymY_F | atcatgcgcataccagtagc | 20 | *rps15-ycf1* |
| CymY_R | agctactatgttcacagaagca | 22 |  |
| CymR_F | ggcaattgtgaactctcggg | 20 | *rpl32-trnL-UAG* |
| CymR_R | tcttcctcagtagctcagcg | 20 |  |

**Table S4.** The list of sample numbers of the samples used in the validation of DNA barcodes.

| Species | Sample No. | Location collected | Longitude | Latitude | Altitude |
| --- | --- | --- | --- | --- | --- |
| *Cymbaria mongolica* | Cm1 | Binzhou, Shaanxi Province, China | 108°13′06.11″E | 35°14′38.63″N | 1035.3 m |
|  | Cm2 | Helan, Ningxia Hui Autonomous Region, China | 105°59′35.67″E | 38°42′05.28″N | 1357.6 m |
|  | Cm3 | Helan, Ningxia Hui Autonomous Region, China | 105°59′35.67″E | 38°42′05.28″N | 1357.6 m |
|  | Cm4 | Tianzhu, Gansu Province, China | 103°04′44.68″E | 37°01′12.47″N | 2552.6 m |
|  | Cm5 | Tianzhu, Gansu Province, China | 103°04′44.68″E | 37°01′12.47″N | 2552.6 m |
|  | Cm6 | Xining, Qinghai Province, China | 101°49′33.59″E | 36°34′13.16″N | 2274 m |
|  | Cm7 | Xining, Qinghai Province, China | 101°49′33.59″E | 36°34′13.16″N | 2274 m |
| *Cymbaria_daurica* | Cd1 | Xilingol, Inner Mongolia Autonomous Region, China | 116°47′07.76″E | 43°28′13.92″N | 1320.7 m |
|  | Cd2 | Yanqing, Hebei province, China | 115°57′34.71″E | 40°22′42.65″N | 565 m |
|  | Cd3 | Yanqing, Hebei province, China | 115°57′34.71″E | 40°22′42.65″N | 565 m |
|  | Cd4 | Yijinhuol Banner, Inner Mongolia Autonomous Region, China | 110°11′42.41″E | 39°33′08.35″N | 1372 m |
|  | Cd5 | Yijinhuol Banner, Inner Mongolia Autonomous Region, China | 110°11′42.41″E | 39°33′08.35″N | 1372 m |
|  | Cd6 | Daqing, Heilongjiang province, China | 124°32′09.86″E | 46°23′51.56″N | 159 m |
|  | Cd7 | Daqing, Heilongjiang province, China | 124°32′09.86″E | 46°23′51.56″N | 159 m |

**Table S5.** Estimated mean age, 95% highest posterior density (HPD) interval, and posterior probability (PP) value of calibrated and key nodes.

| Nodes | Calibration ages (Mya) | Mean age (Mya) | 95% HPD (Mya) | PP |
| --- | --- | --- | --- | --- |
| C1: Orobanchaceae crown | 56 ± 10 | 49.96 | 28.87 - 70.85 | 1.00 |
| Parasites crown | / | 42.95 | 24.61 - 62.40 | 1.00 |
| C2: Pedicularideae crown | 35 ± 10 | 23.11 | 10.07 - 38.44 | 1.00 |
| Cymbarieae crown | / | 31.44 | 14.20 - 50.53 | 1.00 |
| *Cymbaria* crown | / | 6.72 | 01.04 - 16.51 | 1.00 |
